# Carbon monoxide separation: past, present and future

**DOI:** 10.1039/d3cs00147d

**Published:** 2023-04-21

**Authors:** Xiaozhou Ma, Jelco Albertsma, Dieke Gabriels, Rens Horst, Sevgi Polat, Casper Snoeks, Freek Kapteijn, Hüseyin Burak Eral, David A. Vermaas, Bastian Mei, Sissi de Beer, Monique Ann van der Veen

**Affiliations:** a Chemical Engineering Department, Delft University of Technology van der Maasweg 9 2629 HZ Delft The Netherlands m.a.vanderveen@tudelft.nl +31 15 2786458; b Science and Technology Faculty, University Twente Drienerlolaan 5 7522 NB Enschede The Netherlands; c Process & Energy Department, Delft University of Technology Leeghwaterstraat 39 2628 CB Delft The Netherlands; d Chemical Engineering Department, Marmara University 34854 İstanbul Turkey; e Industrial Chemistry, Ruhr-University Bochum Universitätsstr. 150 44801 Bochum Germany

## Abstract

Large amounts of carbon monoxide are produced by industrial processes such as biomass gasification and steel manufacturing. The CO present in vent streams is often burnt, this produces a large amount of CO_2_, *e.g.*, oxidation of CO from metallurgic flue gasses is solely responsible for 2.7% of manmade CO_2_ emissions. The separation of N_2_ from CO due to their very similar physical properties is very challenging, meaning that numerous energy-intensive steps are required for CO separation, making the CO separation from many process streams uneconomical in spite of CO being a valuable building block in the production of major chemicals through C1 chemistry and the production of linear hydrocarbons by the Fischer–Tropsch process. The development of suitable processes for the separation of carbon monoxide has both industrial and environmental significance. Especially since CO is a main product of electrocatalytic CO_2_ reduction, an emerging sustainable technology to enable carbon neutrality. This technology also requires an energy-efficient separation process. Therefore, there is a great need to develop energy efficient CO separation processes adequate for these different process streams. As such the urgency of separating carbon monoxide is gaining greater recognition, with research in the field becoming more and more crucial. This review details the principles on which CO separation is based and provides an overview of currently commercialised CO separation processes and their limitations. Adsorption is identified as a technology with the potential for CO separation with high selectivity and energy efficiency. We review the research efforts, mainly seen in the last decades, in developing new materials for CO separation *via* ad/bsorption and membrane technology. We have geared our review to both traditional CO sources and emerging CO sources, including CO production from CO_2_ conversion. To that end, a variety of emerging processes as potential CO_2_-to-CO technologies are discussed and, specifically, the need for CO capture after electrochemical CO_2_ reduction is highlighted, which is still underexposed in the available literature. Altogether, we aim to highlight the knowledge gaps that could guide future research to improve CO separation performance for industrial implementation.

## Abstract 2

This review discusses the developments in CO separation techniques in the last few decades and the gaps in knowledge it leaves, as well as sources of CO, both traditional and emerging, which would benefit from improved CO separation.

## Graphical abstract

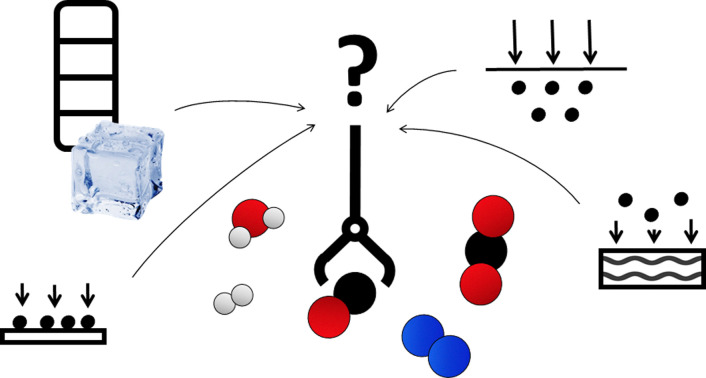

## 1 Introduction

**Fig. 1:**
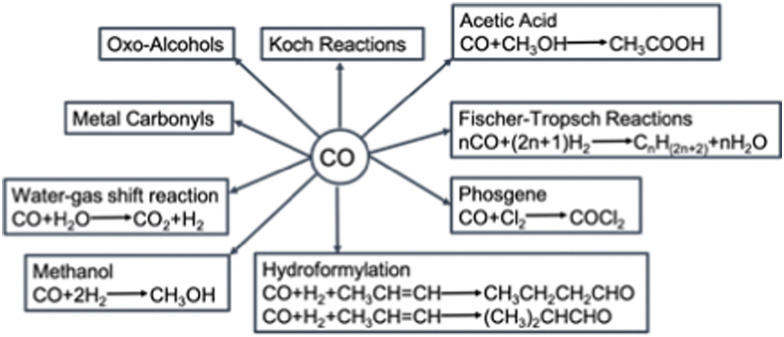
The utilisation of CO in chemical industry.

Carbon monoxide (CO) is an important component for chemical reactions such as the water–gas shift, Fischer–Tropsch, methanol synthesis, steel making in blast furnaces and the Koch reaction. Additionally, it is a key reactant for the synthesis of various organic chemicals (Fig. 1), *e.g.*, light hydrocarbons fuels, methanol, acetic acid, metal carbonyls, phosgene and (oxo-alcohols).^1^ Besides the use of CO as feedstock for the chemical industry, CO can be employed as a direct product as well. For example, in food packaging^2^ and as a therapeutic agent in clinical applications.^3,4^ For these applications, a CO gas stream of either high purity (>99 mol%) or in a well-defined ratio with other gases is needed. These CO gas streams are currently produced by energy intensive processes such as steam methane reforming or autothermal reforming of methane, and consecutive purification.

Next to being an important feedstock, CO is a common by-product. Large amounts of CO are produced during steel manufacturing, petroleum refining or fossil fuel combustion. When captured, this CO could be a copious resource. However, the manufactured CO is usually present in a complex mixture with other gases (*e.g.*, CO_2_, CH_4_, N_2_, H_2_, H_2_S and other sulfur compounds, *etc.*), which makes the separation of CO energy and cost intensive. Especially the separation of N_2_ and CO is not straightforward, due to the very similar physical properties of these molecules regarding molecular mass, size and boiling point, such that classical separation technologies cannot be utilised. This has hampered the reuse of CO as a resource material.^5^ Instead, the CO-containing mixtures are currently burnt for their caloric value, while producing significant quantities of CO_2_. Thus, by developing economically viable separation technologies that allow for reuse of the CO byproduct, important feedstock is generated, and CO_2_ emissions are reduced. The development of these separation technologies is, however, challenging.

Given the intrinsic limitations for separating CO based on weight, size or boiling point, researchers are designing new materials and processes that enable separation by affinity-based adsorption.^6–8^ The development of adsorption-based CO separation technologies will not only be key in the transition to a circular economy, it can be utilised in other applications as well, such as in sensing^9^ or in emerging technologies such as electrocatalytic and photocatalytic reduction of CO_2_. In the latter, the continuous separation of CO will likely contribute to the yield and overall efficiency of the process, which in-turn will further promote establishing a carbon neutral society.^10^ Recently NASA developed a new instrument, called the MOXIE, which can convert carbon dioxide into oxygen and carbon monoxide to provide enough breathable oxygen on Mars and to make fuel for the return journey to earth.^11^ For safety reasons, these products need to be carefully separated and CO adsorbents can capture the last traces of CO. Thus, all in all, the separation of CO has a profound significance.

The present article aims to give a holistic review on the current state-of-the-art of different technologies for CO separation. Although a great deal of research has been conducted, an in-depth overview of the current progress in the field is still missing. Previous reviews concerning CO adsorption have either covered numerous related adsorbates (*e.g.*, different types of gases to separate)^6^ or only focused on a single type of solid adsorbent (*e.g.*, metal–organic frameworks)^7,8^ or more comprehensive, but with a large focus on dispersion of CuCl on porous support.^12^ Our review aims to take a rounded analysis and systematic comparison of the different separation techniques for CO, comparing the different types of commercialised CO separation techniques (Section 2) with the current state-of-the-art is research on CO separation *via* absorption, adsorption and membrane separations (Section 3). *Via* this comparison and by discussing the separation needs of existing CO-containing streams, as well as emerging CO streams (*e.g.*, *via* electrocatalytic reduction of CO_2_), we can pinpoint current research gaps, and make recommendations. We anticipate that this review will provide inspiration for future discoveries of solid CO adsorbents, with a focus on the needs of future technologies.

## 2 Background (Industrial technologies for CO separation)

### 2.1 Physico-chemical properties of CO

Separation methodologies are primarily based on differences in physico-chemical properties between components. Common industrial examples for gas separations include differences in relative volatility seen in distillation, the interaction strength or binding energy in adsorption, and solubility in absorption and membrane separation. For CO, these separation processes can incur significant costs as some gases are difficult to separate from CO. To understand the nature of this difficulty, some physical and chemical properties of CO in relation to other gas molecules are illustrated below.

Under normal atmospheric conditions, CO is a colourless, tasteless and odourless gas. The CO molecule consisting of one carbon atom and one oxygen atom, yielding a total of 10 valence electrons. Those electrons are distributed as defined by the octet rule, where four shared electrons originate from the oxygen atom and two from the carbon atom. The resulting triple bond consists of 2 pi-bonds and 1 Sigma bond that is sp-hybridised, with a length of 112.82 pm^13^ and a bond association energy of 1.070 MJ mol^−1^.^14^ Two electrons derived from the oxygen atom in one of the bonding orbitals form a dipolar bond, causing polarisation within the molecule. Moreover, the difference in electronegativity between the carbon and oxygen atom, 2.55 and 3.44 respectively on the Pauling scale,^15^ leads to an unequal charge distribution within the molecule. These two characteristics and the asymmetric geometrical nature of the CO molecule, together, give rise to a relatively small electric dipole moment. The solubility of CO in water is very poor and becomes almost negligible above 340 K. It is, however, easily soluble in compounds like chloroform, ammonium hydroxide, benzene, and ethanol.

Table 1 provides an overview of the physical property data of CO and components commonly found in CO containing gas mixtures. It follows from the data that separation of CO from H_2_ and CO_2_ should be feasible based on significant differences in boiling point or the kinetic diameter. However, the separation of CO from N_2_ will be more challenging as their boiling points (81.6 K *vs.* 77.4 K) and kinetic diameter (3.76 Å *vs.* 3.64 Å) hardly differ. Separating these two gases based on physical principles would require extreme process conditions to reach the desired gas purity, though separations based on the difference in dipole and quadrupole moment are feasible, as well as on their difference in electronic structure (see Section 2.2). The choice of separation principle is clearly constrained by the gas composition.

**Table 1:** Physical property data ref. 16–18

Compound	Molecular weight [g mol^−1^]	Kinetic diameter [Å]	Normal boiling point [K]	Density (298 K, 1 atm) [kg m^−3^]	Polarisability [10^25^ cm^3^]	Dipole moment [D]/[3.336 × 10^30^ C m]	Quadrupole moment [10^20^ C m^2^]
CO	28.02	3.69	81.66	1.145	19.5	0.122	8.35
CO_2_	44.01	3.30	216.55	1.808	26.5	0	14.4
H_2_O	18.01	2.65	373.15	997	14.5	1.855	—
H_2_	2.02	2.827–2.89	20.27	0.089	8.042	0	2.2
N_2_	28.01	3.64–3.80	77.35	1.1606	17.407	0	5.08
CH_4_	16.04	3.758	111.66	0.657	25.93	0	0
H_2_S	34.08	3.623	212.84	1.36	37.82–39.5	0.978	—

### 2.2 The mechanisms of CO absorption and adsorption

Adsorption and absorption separation processes are based on the difference in interaction energy or binding energy on a surface or in a solvent. Commonly two types are distinguished: physical and chemical absorption, and for adsorption, physi- and chemisorption.

#### 2.2.1 Physical absorption and physisorption

Physical absorption and physisorption are the ab/adsorption processes defined by weak physical interactions which only slightly perturb the electronic structure of the ab/adsorbate, which means there is no chemical bonding.^19^ Examples of these weak non-covalent interactions are van der Waals forces and π–π interactions. Both of these interactions are possible with CO: van der Waals forces are universal and CO is known to interact with aromatic rings through π–π interactions.^20^ The heats of physisorption and physical absorption range from −10 to −40 kJ mol^−1^. These processes usually follow Henry's law, meaning that the temperature dependent equilibrium concentration is proportional to the partial pressure, and the ab/dsorbate does not react with the solvent or adsorbents. Because uptake is higher at high partial pressures, pressurising of the feed is the main source of energy consumption, making physical absorption or physisorption generally uneconomical when ab/dsorbate feed concentration is under 15%.^21^ To regenerate the loaded ab/adsorbent, heat is provided, pressure is reduced or a combination of both.

#### 2.2.2 Chemical absorption and chemisorption

Chemical absorption and chemisorption are characterised by changes in the electronic structure of bonding atoms or molecules and results in a chemical interaction between the ab/adsorbent and the ab/adsorbate. The bonds can be covalent, coordinative or ionic in character. The heats of chemisorption range from −80 to −400 kJ mol^−1^. These processes are preferred when the ab/adsorbate concentration or partial pressure in the feed is low. For chemical absorption, the liquid phase is chosen in a way that the equilibrium favours the formation of the intermediate complex. Two types of chemical absorption are distinguished: reversible and irreversible absorption. In reversible absorption the solvent can be regenerated using heat or pressure changes, in the second case the bonded intermediate is essentially irreversible and is commonly applied for trace amounts to be absorbed and where marginal exit concentrations are required. In chemisorption and chemical absorption, the ad/absorption rate can be slow, leading to a long time to reach the uptake equilibrium. The rate increases with an increase in temperature, but often disfavours the equilibrium uptake.

**Fig. 2:**
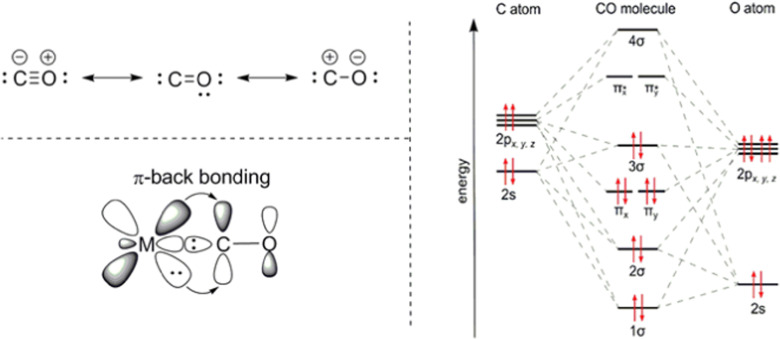
Electronic structure of CO and its binding scheme with transition metals. The π-backbonding occurs due to the donation of d-orbital electrons to the π*-orbitals of CO, π_x_* and π_y_*. Reprinted from ref. 23, Copyright (2019) with permission from Elsevier.

Chemisorption and chemical absorption are based on the chemical interaction of the target molecule and the ad/absorbent determined by the electronic structure of both. Therefore, the electronic structure of CO is essential for its binding mechanism to metals and metal ions^22^ (Fig. 2). In CO, the oxygen atom supplies four electrons to the C–O triple bond, causing an uneven electron density distribution in the molecular orbitals. The oxygen becomes slightly positively charged, and the carbon slightly negatively charged. The HOMO−1 molecular π bonding orbitals of CO (π_*x*_ and π_*y*_) are concentrated on the oxygen atom, while the HOMO bonding σ_C_ orbital as well as the LUMO antibonding π* orbitals (π_x_* and π_y_*) are concentrated on the carbon atom. The electronic structure causes the preference of binding through the carbon atom onto metals, as the σ_C_ orbital can form a σ-bond with one of the empty d-subshell orbitals of the metal through electron donation. Furthermore, the metal can also back-donate electrons from its d-orbital to the π* antibonding orbitals of CO perpendicular to the bond axis. This back-donation weakens the internal C–O bond, due to the filling of the antibonding π* orbitals, but strengthens the bond between the metal and CO.

##### 2.2.2.1 Spin crossover

**Fig. 3:**
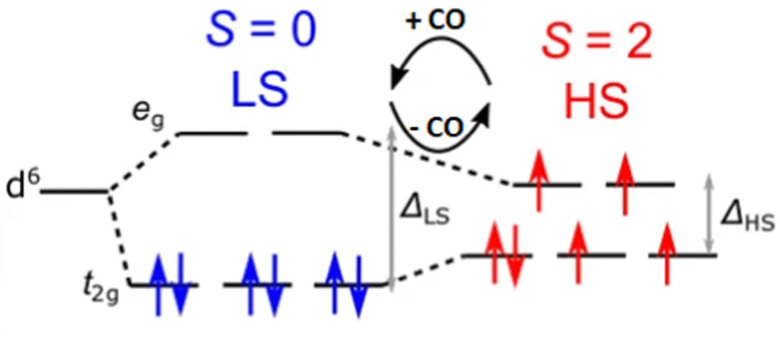
Schematic of the spin crossover effect caused by the adsorption of CO onto unsaturated octahedral d^6^ metal sites. Figure reprinted from ref. 25 with modifications with permission from Springer Nature, copyright 2015.

An effect that can be utilised to influence the chemisorption of CO is spin crossover (Fig. 3). When ligands are bound to metal atoms, the symmetry of the d-orbitals is broken due to interaction with said ligands. This symmetry breaking causes a change in the energy of the individual d-orbitals, resulting in a splitting of the levels. Depending on the number of ligands and the symmetry of the ligands around the metal, several level splittings can occur, such as octahedral and tetrahedral level splitting. If a metal contains four to seven electrons in its d-subshell multiple spin states can exist due to the allocation of electrons to the various levels. The metal can either be in a low spin state in which the total spin of the complex is minimised or in a high spin state in which the total spin is maximised. Which spin state is the most stable depends on the magnitude of the splitting parameter and the energy penalty for pairing two electrons in the same d-orbital. The splitting parameter itself depends on the type of metal and its charge, as well as the ligands surrounding the metal. A crowded metal ion will generally favour a high spin configuration as indicated by high spin metal ions having higher metal–ligand bond lengths.^24^ When the difference in energy between the splitting energy and the pairing energy is small, spin crossover can occur wherein the complex can change between spin states. Generally, such spin crossover effects are observed in octahedral complexes as therein the splitting parameter is larger, allowing for a more accessible low spin state.

This spin crossover can be utilised to influence the chemisorption of CO. CO can accept electrons through its π* antibonding orbitals which are higher in energy than the d-orbitals of the metal. This electron accepting character of CO will increase the splitting parameter if it chemisorbs to a metal. This increase in the splitting parameter can lead to a change from the high spin state to the low spin state resulting in a stronger CO bond.

### 2.3 Heme binding of CO and the influence of water on CO adsorption

Many of the process streams from which CO is to be separated are humid streams. Especially in the case of chemisorption and chemical absorption, water can cause instability of the CO-metal ion complex (see Section 4.1), meaning this humidity needs to be removed before the separation process, which is economically unfavourable. In this regard, the binding of CO to hemoproteins, which happens in aqueous conditions, deserves consideration.

The high toxicity of CO, causing the dysfunction of hemoproteins (*e.g.*, hemoglobin, myoglobin and cytochrome *c* oxidase) that are indispensable for carrying or consuming O_2_, is due to the formation of a stable complex called carboxyhemoglobin. This complex is over 200 times stronger than the hemoglobin complex with oxygen,^26^ which causes a deficit of oxygen in the tissue of the exposed organism over time.

In the carboxyhemoglobin complex, the binding with CO distorts the d orbitals of the Fe^2+^. The CO binds to a site in line with the *z*-axis. The original t_2g_ orbitals, d_*xy*_, d_*xz*_, and d_*yz*_, are split in energy due to the stabilising of the d_*xz*_ and d_*yz*_ orbitals, while the e_g_ orbitals remain at the same energy level. These new levels are all concentrated on the metal centre, while the π* orbitals increase in energy and are concentrated on the CO ligand. As already established, the σ_C_ molecular orbital forms a bond with a d-orbital from the metal, d_*z*^2^_ in the example with the CO in line with the *z*-axis, which is called a donation interaction. However, the metal can also back-donate electrons to CO into the empty π* orbitals. These π–interactions strengthen the M–C bond while simultaneously weakening the C–O bond due to the filling of the antibonding orbitals. Due to π-back bonding, the carbon atom of CO interacts strongly with Fe(ii) in hemoproteins with high stability. What is even more interesting is that the reversible CO binding in hemoproteins can hardly be impacted by the presence of water, which is rarely seen in CO adsorbents. The reason is that the native hemoproteins protect the heme site with apolar amino acids which form a solvent penetration barrier, reduce the size of the cavity, and form a hydrophobic pocket which reduces the occurrence of the water-catalyzed autoxidation of the Fe(ii) to Fe(iii) porphyrins.^27^ On top of these protection mechanisms, it was also found that the binding energy of water with Fe–porphyrin–imidazole, a complex which is used as a simple model for the heme site, was found to be more than 20 kJ mol^−1^ lower than that the binding of the same complex with CO,^28^ making CO the statistically more likely adsorbate. Perhaps development of new CO capture approaches can take a clue from nature. For instance, haemoglobin utilises a strategy called allostery to optimise its CO_2_ binding capacity by going through significant conformational changes.^29^ The overarching idea of leveraging stereochemistry of cooperative effects in allostery, higher efficiency CO binding can also be achieved. Notably, a synthetic supramolecular compound composed of an Fe(ii)porphyrin and a cyclodextrin dimer has been demonstrated to reversibly bind to CO at ambient temperatures.^30^

### 2.4 Industrial technologies

In this Section current commercial separation strategies are evaluated and discussed for their application for carbon monoxide capture at industrial scale. Technnologies that are discussed are based on cryogenic distillation, absorption, membrane separation and adsorption.^31^ The most suitable technique is very often dependent on feed conditions and compositions and on the required end-product specifications. The advantages and disadvantages of each technology are introduced and compared.

#### 2.4.1 Cryogenic distillation

Cryogenic distillation is the dominant industrial technology used to obtain pure gas species from gaseous mixtures. The separation process is based on the relative volatilities of the components in the mixture using the effective vapour pressures to extract one or several components from the mixture.^32^ The cryogenic range begins when so-called permanent gases (N_2_, O_2_*etc.*) start to liquefy around −150 °C. The reason to chill gases to these low temperatures is that phase changes start occurring, and separation can be performed based on differences in condensation and sublimation point. Based on this principle, high purity oxygen and nitrogen (>99.9%) are obtained in air separation^33^ and, in a similar manner, CO_2_ can be captured from flue gases with 99.99% purity and recovery.^34^ These examples indicate the possibilities to obtain a high quality product by cryogenic processes.

There are two types of cryogenic process used in commercial industry to separate carbon monoxide from gas mixtures containing H_2_: low-temperature partial condensation cycle and liquid methane wash.^35^ For both cases, traces of contaminants such as water and CO_2_ that could freeze in the unit can cause clogging problems. Hence, they must be eliminated from the feed gas by a pretreatment procedure: the process gas is initially dried in a molecular sieve adsorber station. Separation of CO and N_2_ using a cryogenic distillation process is not performed since these two gases have a close boiling temperature (CO: 81.55 K; N_2_: 77.35 K at atmospheric pressure).^36^

##### 2.4.1.1 The low-temperature partial condensation process

**Fig. 4:**
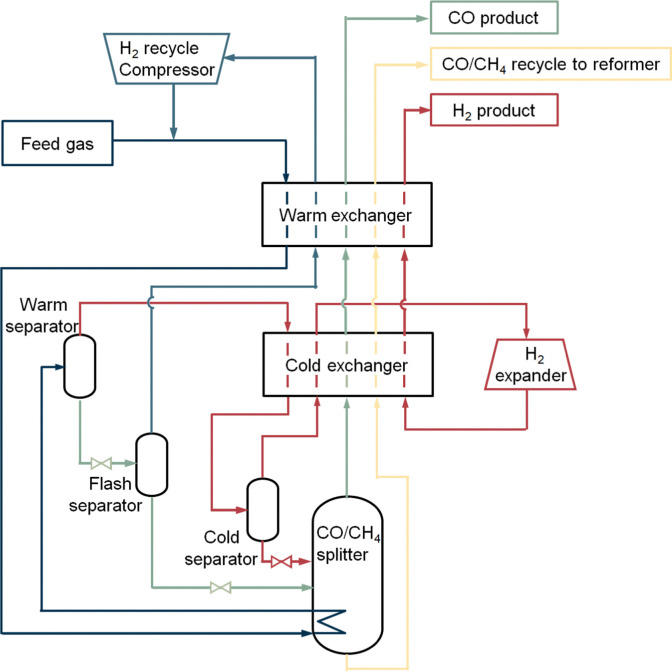
Low-temperature partial condensation process. Figure based on process scheme from Dutta *et al.*^31^

The partial condensation cycle operates with gas mixtures (*e.g.*, from partial oxidation) supplied at high pressure, with high CO, H_2_ and low CH_4_ concentration.^35^ This process utilises the difference in the boiling points of the main components of the feed gas. In the separation procedure (Fig. 4) the feed gas first enters a warm exchanger and is cooled against the cold product stream. It is further cooled down by using its heat to reboil the CO/CH_4_ splitter. In the warm separator, the liquefied CO and CH_4_ are separated from the hydrogen vapour. The separated hydrogen vapour is cooled down in the cold exchanger and enters a cold separator. In this separator most of the remaining CO in the hydrogen vapour will be condensed and separated from the vapour. The CO liquid flow is used as a reflux in the CO/CH_4_ splitter. The H_2_ vapour collected overhead from this cold separator flows through the cold exchanger to be heated up. After that it is cooled down by expansion and then warmed up in both the cold and warm exchanger by absorbing the heat. The hydrogen vapour product recovered from the process has a purity of 98%. The CO and CH_4_ containing liquid from the bottom of the warm separator are reduced in pressure and dropped in temperature by a throttle valve. Hereafter, some remaining hydrogen gas is further removed from the CO and CH_4_ liquid stream in the flash separator. The hydrogen vapour from the flash separator is rewarmed, compressed, and recycled back to the incoming feed to recover more CO from the stream. The liquid from the flash separator containing mostly carbon monoxide and methane flows into the CO/CH_4_ splitter, which acts as a distillation column, and is rewarmed. Carbon monoxide vapour flows out of the splitter and warmed up in both exchangers with a purity of 98–99%. In order to use CO in a downstream process, compression of the CO product stream is necessary. The remaining liquid containing methane and carbon monoxide leaves the splitter through the bottom, whereafter it is heated up and exit the process as a reformer fuel.

The partial condensation cycle process has a carbon monoxide recovery of 75–80%. Due to uncondensed carbon monoxide in hydrogen vapour and leftovers in the methane flow, a 100% yield is not reached. The total capacity of the system is 55 000 N m^3^ h^−1^ product CO. The operating expenditures (OPEX) to keep the process running is 18–100 kW h per tonne CO product. Most energy is consumed by the pretreatment, pressurisation of the feed, H_2_ recycle compressor and the H_2_ expander. In addition, it may be assumed that some energy is used to provide extra cooling in case the refrigeration system falls short. Other costs are dependent on the quality of the feed gas, the required purity and pressure of the CO product.

##### 2.4.1.2 The liquid methane wash process

The liquid methane wash process operates with low pressure feed gases, for instance gas mixtures from steam reforming. The feed gas usually contains a low CH_4_/CO ratio and a high CH_4_ concentration. The feed gas is pretreated by removing residual water and CO_2_ before entering the system with a pressure of approximately 1.5–3.5 MPa.^32^

**Fig. 5:**
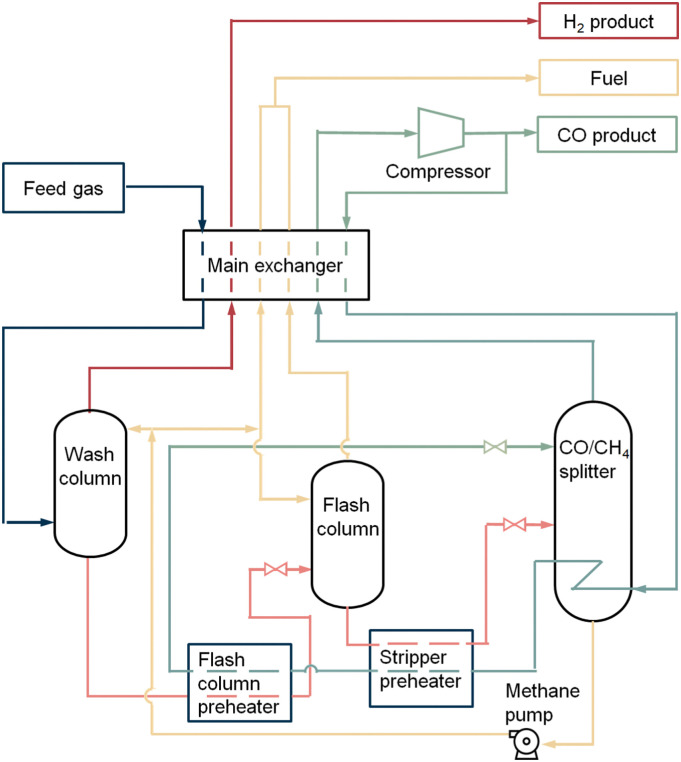
The liquid methane wash process. Figure based on process scheme from Dutta *et al.*^31^

Fig. 5 is a representation of a liquid methane wash process. The feed gas is cooled down through the main exchanger to a temperature of 93 K and enters the wash column at the bottom. CO and CH_4_ are liquefied through this process, while the hydrogen remains as vapour. To prevent losses of carbon monoxide in the hydrogen vapour, the rising hydrogen gas is washed with a cooled methane refluxing in the wash column. This liquid methane reflux enters the column *via* the top and washes the carbon monoxide out of the hydrogen gas. The hydrogen vapour is heated up in the main exchanger and leaves the system as a product with a purity of 99% (with small amount of methane (1–1.5%)). The liquid containing mostly CO and CH_4_ flows out of the wash column, whereafter it is preheated immediately followed by cooling down the dissolved gas through expansion in the flash column. The generated vapour leaving this column, consisting of hydrogen dissolved in methane, is rewarmed and recovered as a fuel product. Similarly, a cooled methane reflux enters into the flash column and washes the gas vapour to reduce the carbon monoxide loss. The liquid from the flash column, which is free of hydrogen, is heated and flashed into the warmed CO/CH_4_ splitter. Carbon monoxide vapour flows out of the splitter at the top. After this it is rewarmed and compressed. A part of the stream will leave the system as a CO product with a purity of 99%, the other part will be cooled and flows back as a recycle stream. This recycle stream firstly reboils the CO/CH_4_ splitter and warms up the heaters, secondly it is cooled down by expansion in a throttle valve. The carbon monoxide condensate is used as a reflux in the splitter. The methane liquid in the splitter is pumped into the wash and flash column to serve partly as cooled reflux stream. The remaining methane liquid is heated up and leaves the system as fuel gas.

The liquid methane wash cycle has a high carbon monoxide recovery of 99%. The total capacity of the system is 34 000 N m^3^ h^−1^ product CO. The OPEX in this process is 300–600 kW h per tonne. The energy is consumed by the pretreatment, pressurisation of the feed, preheaters, the CO product compressor, methane pump and extra cooling for the system if the refrigeration system falls short. Quality of the feed gas, purity and pressure of the CO product determine the other economic costs.

##### 2.4.1.3 Concluding remarks

The advantages of cryogenic distillation are well-known, some include high maturity of the process, no need to introduce additional components and the ability to produce high purity products combined with minimal losses of product.^37^

Although this technology has the advantage of economy of scale, capital and energy costs associated with cryogenic distillation for CO separation are significant. The need to operate at very low temperatures lead to the inherent need for cooling, incurring additional compression power costs for refrigeration compressors. An expensive external cooling utility, such as liquid nitrogen is also required to satisfy the process cooling demands, further increasing operating costs. In addition, to meet the requirement of operating at harsh conditions, the investment of constructing the distillation columns and auxiliary units is much higher than those operated at ambient temperature and low pressures. Finally, it is important to note that, due to the close boiling points of CO and N_2_, it is impossible to recover pure CO from gas-mixtures that contain N_2_ using a cryogenic distillation process.

#### 2.4.2 Absorption

Absorption separation is based on relative solubilities of individual components in the mixture in liquid phase, meaning some components get enriched in the bulk aqueous phase, while the remainder stays in the gas phase, leading to a separation of the mixtures.

Only chemical absorption technology is reported for CO absorption, which uses chemical solvents that react reversibly with the gas component.^31^ The CO absorption separation processes, such as COSORB, COSORB^II^ or COPure^SM^, based on CuAlCl_4_ complexes in toluene operate with different kinds of feed gases, for example refinery gas, coke oven gas, blast furnace gas from the steel industry, steam reforming gas and a few more. The process can separate CO from feed gases that contain N_2_, CO_2_, H_2_ and CH_4_. The gas mixtures need to be pretreated if they contain impurities such as water and gases with sulfur components. Water can degrade CuAlCl_4_ into HCl, which leads to the corrosion of the steel columns. H_2_S and other sulfur compounds could lead to the precipitation of Cu(i) sulfide. Molecular sieve adsorption and refrigeration are used for removal of water. Activated carbon adsorption is used to remove sulfur components.

##### 2.4.2.1 COSORB process

**Fig. 6:**
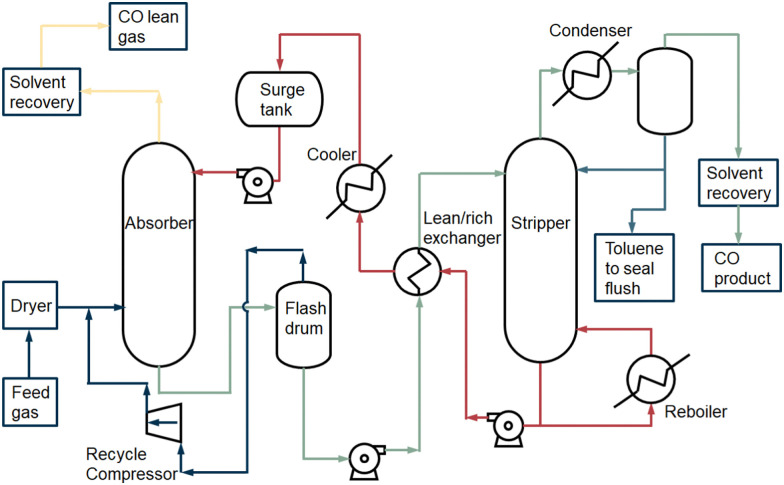
The basic COSORB process. Figure based on process scheme presented in Keller *et al.*^38^

The general COSORB process which is developed by Tenneco Chemicals is displayed in Fig. 6.^38^ In this process, the cuprous aluminium chloride (CuAlCl_4_) complex is utilised to selectively capture carbon monoxide over other gases (*e.g.*, CO_2_, H_2_, N_2_ and CH_4_) in a toluene solution. The stoichiometric reaction can be described as follows:
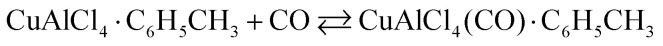


The activation energies evaluated for the forward and reverse reactions were −32.93 and −97.42 kJ mol^−1^, respectively.^39^ The chemical CO absorption mechanism is based on the interaction between the CO gas molecule and the Cu(i) metal ion. The σ orbital of the carbon site in CO donates electrons to the empty d-orbital of the copper atom and forms a σ bond. Meanwhile, the electrons in a filled d-orbital of the copper move to an empty π*-orbital of the CO molecule and a strong π-back bonding is formed.

The feed gas^38^ flows into the absorber column after the pretreatment. It contacts with the CuAlCl_4_ in toluene in counter current flow. A CO complexing compound is formed. In addition, CO_2_, H_2_ and CH_4_ are physically absorbed in toluene. Vaporised toluene is recovered from the overhead gas through compression and adsorption. A part of the physically absorbed gas components in toluene also leaves the process *via* the top of the absorber column. The liquid, containing chemically absorbed CO and physically dissolved CO_2_, H_2_ and CH_4_, is separated in the flash drum by a drop in pressure. The solution is then decompressed to release the physically dissolved gases (CO_2_, H_2_ and CH_4_), which can be recycled back to the absorber column. The CuAlCl_4_(CO) complex solution is heated up in the heat exchanger with the counter current stream which is derived from the bottom of the stripper. The solution enters the stripper, and the carbon monoxide is decomplexed from Cu(i) by heating the solution at high temperature (408 K) and lowering the pressure to *ca.* 0.15 MPa.^5^ The carbon monoxide flows out at the top of the stripper and is cooled. The co-evaporated toluene in the CO stream is separated. The recovered toluene either flows back into the splitter or is sealed and removed from the process. Before the carbon monoxide vapour flows out of the process, the remaining toluene, which is volatile and harmful, is extracted from the product. The CO product stream is compressed in order to be used in a downstream process. A part of the solvent with CuAlCl_4_ dissolved in toluene from the bottom of the stripper is reused in the stripper. The remaining part flows back to the absorber column and heat is recovered in the lean/rich heat exchanger. To recover a pure CO product after absorption to the CuAlCl_4_, decomplexation takes place at low pressure and high temperature.^40,41^

The CO product capacity of COSORB absorption separation units ranges from 270 to 5500 N m^3^ h^−1^. The final recovery of CO is 98% and typical purity is 99% for CO feed concentrations >0.16 kmol m^−3^.^42^ To run the process the utility consumptions are 392 kW h per tonne product for electrical power (climate-depending) and 1448 kW h per tonne CO for cooling water and reboiler heat.

COPure^SM^ is the commercial process used today for CO absorption, which is an upgrade of old COSORB and COSORB^II^ techniques and developed by COSTELLO. In this process, a more effective pretreatment is used and a CO-recovery rate of 98% with a purity of 99% is obtained.

##### 2.4.2.2 Concluding remarks

High purity and recovery of CO can be achieved in an absorption separation process, although there are several disadvantages of the technology: the CuAlCl_4_ complex can be easily poisoned by H_2_O, H_2_S, SO_2_ and oxides of nitrogen, so a pretreatment procedure is required to remove these components. While the energy consumption is very steep: *e.g.*, for COSORB *circa* 400 kW per tonne product for electrical power and *circa* 1400 kW h per tonne CO for cooling water and reboiler heat is required,^43^ absorption is capable of separating N_2_ and CO. On top of that, it has an advantage in capital investment (48% less cost) compared to cryogenic distillation of the most favourable separation of CO and H_2_, and in contrast to cryogenic distillation, CO_2_ does not have to be removed prior to the CO separation.^44^ Meanwhile, the solvent degradation and the precipitation of solids need to be considered. Some environmental and safety issues arise during the absorption process, such as the disposal of spent solvent and handling of the corrosive or volatile solvent. The absorbent degradation is the reason that the COSORB process is relatively seldom used.^31^

#### 2.4.3 Membrane separation

The third technology that is considered is the membrane separation of carbon monoxide. A membrane can be seen as a selective barrier, under influence of a pressure difference as a driving force it allows some species in the gas and liquid phase to pass through, but rejects or retards others. For the separation of CO, only gas membrane separation is relevant. Gas membrane separation is based on the relative permeability of an individual gas through a membrane based on the physical or chemical properties of the gas. The main driving force in the separation is the pressure difference across the membrane and based on the gaseous solubility and permeability, the permeable molecules (permeate) diffuse through and the non-permeable molecules (retentate) stay on the feed stream side. The specific kind of membrane (glassy or rubbery polymers) that is used, is of great importance in the design of a membrane-separation unit as it will hugely affect the performance. Although a lot of research is carried out to develop novel membrane materials, only polymeric membranes are used commercially for CO separation at industrial scale. Here, the higher the solubility of a gas component in the polymer matrix, and the higher its diffusion coefficient in the matrix, the higher its permeability.^45^

The commercial application of membranes in CO purification is mainly focused on the separation and purification of CO from syngas, based on the high permeation rate of hydrogen *versus* CO. Also components like CH_4_, CO_2_ and N_2_ have lower permeation rates in polymeric membranes compared to hydrogen, so it is *a priori* difficult to separate them from carbon monoxide using this method. In industrial processes, syngas (H_2_ + CO) is required to make a variety of products, with each product type demanding a specific, optimal ratio of H_2_ to CO in the feed syngas. However, as the H_2_/CO ratio produced depends on the syngas generation process and the hydrocarbon feed, this primary ratio may not be optimal for downstream products, and some H_2_/CO ratio adjustment is needed. Membrane technology is perfectly suited for this, based on the different permeability of the two gases through the membrane. The gas mixture is fed at high pressure to one side of a thin polymer film or hollow fiber membrane unit, yielding a high pressure CO enriched retentate and a low pressure H_2_-rich permeate. Ramírez-Santos *et al.*^46^ compared process designs for blast furnace gas (BFG) treatment based on a H_2_ selective glassy polymer membrane from UBE industries (B–H) with one based on a CO_2_ selective rubbery polymer membrane from MTR (Polaris). For both systems CO recovery can reach 80–90% but purity is limited to *ca.* 30%, indicating the trade-off in membrane separation.

##### 2.4.3.1 PRISM membrane systems

The PRISM membrane separator (based on polysulfone and developed by Air products) can be utilised not only to purify CO from feed streams containing hydrogen and water vapour, but also to change the H_2_/CO ratio in oxo-alcohol synthesis gas to meet the real application requirements.^47^

The separator consists of polymeric hollow fibers that act as a molecular filter. The feed gas (*e.g.*, 48% H_2_, 51% CO, <1% N_2_ and saturated H_2_O) flows into the system with a pressure of 6 MPa. The pressure at the end of the separation system is 150 kPa, which creates a driving force for the separation. The permeated gas molecules (H_2_, water), which have higher permeation rates compared to carbon monoxide, permeate across the thin skin of the hollow fiber wall driven by a partial pressure difference and are channeled into the permeate stream. The retentate leaves the system with a CO product purity of 85 mol% and a recovery of 85 mol%.^48^ A two-stage system and a recycle compressor can be used to improve the separation and the CO purity can be increased to ≥95 mol%.

##### 2.4.3.2 Cellulose acetate membrane

Toshiba corporation developed a cellulose acetate membrane separation technique to change the H_2_/CO ratio of syngas. This membrane can also be used to produce a CO product stream of 2200 N m^3^ h^−1^ from synthesis gas.^49^

The separation process consists of a two-stage membrane system. In the first stage, the feed enters the system at a pressure of 2.8 MPa and a temperature of 313 K. The hydrogen gas permeates the membrane and flows out of the system with a pressure of 70 kPa. The residue gas leaves the first membrane and enters to the second one with a pressure of 2.7 MPa. The permeate from the second membrane is recycled in order to obtain a high recovery of CO. The non-permeated CO product has a purity of 98%. The cellulose acetate membrane has a high permeability, so less surface area is needed for separation. The sheet-like membrane is configured into spiral wound elements, which is less expensive than hollow fiber modules. Compared to the polysulfone membrane, the cellulose acetate was found to provide higher selectivity and permeability at the same operation conditions for both syngas ratio adjustment and CO production.^31^

##### 2.4.3.3 Concluding remarks

The general advantages of membrane processes include its simple operation and easy (linear) up or down scaling. Meanwhile, since it operates under milder conditions, it can offer cost advantages over cryogenic separation, although it has not the economy of scale advantage. It also reduces the environmental impact due to the absence of any chemicals. Weaknesses of this technology are its sensitivity to fouling by impurities, plasticization by feed components, and compaction caused by pressure or temperature effects. Moreover, the technology is mainly used for separating H_2_ from syngas to adjust the H_2_ : CO ratio. Separation of CO from gases like CO_2_, CH_4_, N_2_, which have similar solubility and diffusivity properties in common polymers, remains challenging. Therefore, thorough pretreatment or multistage separation is often needed in order to achieve sufficient high purity and recovery of CO, which is expensive for some applications.^50^

#### 2.4.4 Adsorption

Adsorptive separation is based on differences in gas adsorption on a solid adsorbent, adsorption selectivity.^51^ Depending on the binding strengths of gas molecules with the adsorbent physisorption and chemisorption are distinguished (Section 2.2). Adsorption technology is a cyclic process: firstly, uptake of gas components upon contact with the solid adsorbents occurs under controlled temperature (temperature swing adsorption: TSA), pressure (pressure swing adsorption: PSA; vacuum swing adsorption: VSA), or electric potential (electric swing adsorption: ESA) conditions. In the second step, the adsorbed gas is released, regenerating the laden adsorbent. In TSA, PSA, VSA or ESA processes, the adsorbents are regenerated by heating, reducing the pressure, under vacuum or in a low-voltage electric current condition, respectively. Three commercial PSA plants that are operated for CO separation are discussed in this section. The setup of adsorption processes can differ in numbers of stages and columns depending on the size of the process.

##### 2.4.4.1 COPISA process (physisorption)

**Fig. 7:**
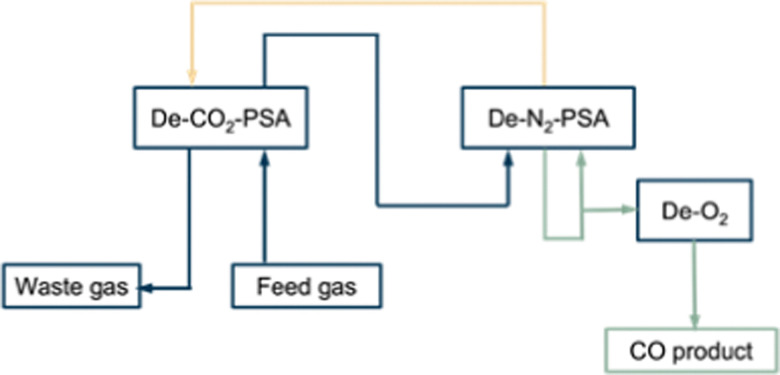
General overview of the COPISA process.

Kawasaki Steel Corporation and Osaka Oxygen Industries LTD developed the first commercial process for CO separation using the PSA technology named COPISA (CO pressure induces selective adsorption). This is a process in which carbon monoxide is recovered from basic oxygen furnace gas (BOF), which is a mixture of CO, CO_2_ and N_2_ (CO: 71%, N_2_: 14%, CO_2_: 13%, O_2_ + H_2_: 2%). The process consists of (Fig. 7): (1) pretreatment process; (2) CO_2_ separation process (De-CO_2_-PSA); (3) N_2_ separation process (De-N_2_-PSA); (4) refining process (De-O_2_ and De-H_2_O). Before the adsorption operation, the feed gas is pre-treated at high pressure to remove the dust followed by compression to 5–15 kPa at 298 K.^52^

In the first section (De-CO_2_-PSA) of the adsorption operation, CO_2_ is removed from the feed gas by an activated carbon adsorbent. De-CO_2_-PSA requires two or three adsorption columns following the process cycles of: adsorption, depressurisation, evacuation, and purge. The CO_2_ is adsorbed whereafter the effluent (mainly CO and N_2_) flows to the next section. The adsorption ends before the CO_2_-breakthrough point at which CO_2_ saturation is reached, to avoid CO_2_ leaking into the effluent gases. For the purge step, a CO_2_-free waste gas from the next section (De-N_2_-PSA) is used. In this section the purge step acts to further desorb and regenerate CO_2_. After evacuation the feed gas slowly pressurises the column up to the required adsorption pressure. After removing CO_2_, the gas effluent leaves the first section and flows to the second section (De-N_2_-PSA). It contains a mixture of 82% CO, 16% N_2_, 2% of H_2_ and O_2_.

In this process a zeolite based adsorbent (Na-MOR) is used. Carbon monoxide physisorbs at a pressure of 150–250 kPa through van der Waals forces with an adsorption capacity of approximately 1 mmol g^−1^ adsorbent (298 K and 1 atm). The CO/N_2_ adsorption selectivity is about 2.5–3.0. The effluent gas originating from the adsorption step, which mainly consist of N_2_ and a small residual amount of CO, is used as a waste gas purge in the De-CO_2_-PSA unit. In the process the vacuum pressure to evacuate CO is set at 6.6–13.3 kPa.

After the adsorption steps, oxygen and water are removed to ensure the utilisation of CO as a raw material in chemical industry. The oxygen is removed by converting it catalytically with CO into CO_2_. Moisture is removed using molecular sieves. The moderate CO/N_2_ ideal selectivity of 2.5–3.0 means that achievement of a high purity of 98% CO, comes at the expense of a lower recovery ratio (45% for a feed with 71% CO, and 60% for a feed with 80–85% CO). Thus, this process is only appropriate for CO-separation from feeds with very high CO concentrations. Unrecovered CO exits the process as waste gas purge. The capacity of the reported process is 410 N m^3^ h^−1^ of CO product. No energy consumption and cost information are disclosed.

##### 2.4.4.2 Activated alumina coated with carbon and impregnated with copper compound (chemisorption)

Kobe Steel Ltd has developed a commercial plant for pressure swing adsorption of CO from Linze-Donawitz converter exhaust gas (LDG: 68% CO, 16% CO_2_, 13% N_2_, 2% H_2_, 1% O_2_ + Ar), however other feed would also be usable as well.^53^ A porous activated alumina coated with a surface carbon layer constitutes the support of the adsorbent. Copper compounds (CuCl, CuCl_2_) dispersed on the support surface provide the active chemisorption sites. The carbon layer serves to prevent the oxidation of Cu(i) and reaction with the alumina, and to reduce Cu(ii) to Cu(i), while the alumina provides the particle texture. Before the gas mixture is fed into the PSA process, it is pretreated in a thermal-swing process over activated carbon to remove sulfur compounds and water. Sulfurous compounds cause the deactivation of the CO adsorbent and water adsorbs strongly to the adsorbent. The PSA process has four adsorption columns, each cycling through four sequential process steps (adsorption, purging, desorption (*ca.* 13 kPa), and repressurisation). In principle, the relatively strong interaction of CO with CuCl already facilitates production of high purity CO (>99%) in a single stage PSA. This is caused by the close to ideal selectivities of *ca.* 25 for CO/N_2_ and 7.5 for CO/CO_2_ (as estimated from the single component adsorption isotherms) at 20 °C. The latter also means that a PSA-stage for the removal of CO_2_ prior to the CO-separation is not necessary. The adsorption capacity for CO at 20 °C is *ca.* 1 mmol g^−1^. Due to the high selectivity for CO a very high purity with high recovery can be achieved: for LDG 99% purity with 90% recovery, and a 99.9% purity with 75–80% recovery. The pilot produces 150 N m^3^ h^−1^ of CO product. Two years of operation showed no deterioration of the adsorbent. Based on the positive result of this plant, Kobe built another plant with a CO product flow of 2000 N m^3^ h^−1^.

##### 2.4.4.3 PU-1 (chemisorption)

The VPSA-CO (VPSA: vacuum pressure swing adsorption) plant, which is developed by Peking University Pioneer Technology Co in recent years, has a CO product flow that varies from 1700 to 20 000 N m^3^ h^−1^. This technology is suitable for any CO-rich feed gas. The separation process consists of two units (VPSA-1 and VPSA-2;). In the first unit, H_2_O, CO_2_ and trace heavy components such as sulfur-containing compounds are removed. This unit contains three adsorption columns, which have low adsorption capacity for CO but high adsorption for CO_2_ and H_2_O. The compressed feed gas enters the VPSA-1 at 801 kPa and room temperature. Each adsorption column undergoes the same PSA steps: adsorption, pressure-equalisation, counter depressurisation, purge and regeneration, partial pressurisation, re-pressurisation. The purge step is done with effluent gas from the VPSA-2 section. The VPSA-2 section provides further purification to obtain a CO product with a purity of 99%. The feed gas enters the VPSA-2 section at 343 K and a pressure of 750 kPa and operates according to the PSA process steps as described before. A vacuum pressure of 13–20 kPa is used in the regeneration step. This section contains four adsorption columns filled with CuCl/zeolite adsorbent, also known as PU-1. It has a CO adsorption capacity of >50 ml g^−1^ equivalent to 2.03 mmol g^−1^ adsorbent at 1 atm and ambient temperature and an ideal CO/N_2_ selectivity at 20 °C of *ca.* 20, with similar values for CO *versus* H_2_ and CH_4_, while the ideal selectivity of CO/CO_2_ is only around 2–2.5. This rationalises why CO_2_ needs to be removed in a first PSA unit (VPSA-1), and why very high purities combined with very high recoveries of CO can be reached. The CO product feed flows out of the process with a purity >99% and a recovery >85% from a syngas gas containing about 30% CO and rich in H_2_ (41%), N_2_ (17%), CO_2_ (8%) and CH_4_ (2%). To keep the process running it requires 340 kW h per tonne CO electricity.^54–56^

##### 2.4.4.4 Concluding remarks

Adsorptive separation of CO, in contrast to cryogenic distillation, does not rely on phase changes and volatility differences to achieve separation. It can be seen as a good candidate for the separation of close-boiling gas mixtures, such as CO and N_2_. It is energy-efficient and has the advantages of low installation and operating costs, low water and electricity consumption, easy operation and maintenance, high production capacity and CO purity, no equipment corrosion and environmental pollution. However, its downsides are: (1) high requirement for raw gas quality: H_2_O or sulfur compounds impurities that reduce capacity need to be removed first; (2) for raw gases with a low CO concentration, the CO recovery rate decreases, especially for the COPISA process based on physisorption; (3) the scale of PSA devices is limited to a certain extent. If the gas volume is large, multiple parallel operation units are required. Meanwhile, the performance of adsorptive separation strongly relies on the adsorption mechanism. Chemisorption performs much better than physisorption in terms of recovery and purity. Yet a chemisorption process has the disadvantage that the energy required for desorption will be larger due to the higher adsorption enthalpies involved. Yet, the PU-1 chemisorption process requires far less energy input than the COSORB process based on chemical absorption (*ca.* 340 *versus ca.* 1400 kW h per tonne CO, respectively), but with some loss in recovery (85% for PU-1 *versus* 98% for COSORB). The choice of a proper sorbent has a significant influence on the CO adsorption performance and process design. With thousands of known adsorbents and novel adsorbents being constantly developed, identifying a suitable adsorbent and separation scheme for a given separation problem remains a challenging task.

#### 2.4.5 Evaluative notes

At present, several technologies are already being used for CO separation. Each of them has pros and cons related to the separation performance (see Table 2). For the purification of syngas and composition adjustment most of the technology is focused on the intrinsic differences in the physical properties between H_2_ and CO. In this case cryogenic distillation, pressure swing adsorption and membrane separation can be readily employed. Cryogenic distillation here has the advantages of being a very established technology that operates favourably at very large scale. Membrane separation on the other hand has the advantages that pretreatment for the removal of H_2_O and CO_2_ is not necessary, and that less energy is required compared to cryogenic distillation. However, there is no economy of scale, which limits the capacity. In fact, all other current CO separation processes require removal of H_2_O *via* pretreatment. Moreover, beyond membrane separation, the only processes that do not require removal of CO_2_, are based on chemical absorption and on chemisorption.

**Table 2:** A comparison of existing industrial CO purification technologies

Separation mechanism	Separation technology	CO product stream			Energy needs [kW h per tonne CO]	Advantages	Limitations
		Purity [%]	Recovery [%]	Throughput [N m^3^ h^−1^]			
Cryogenic distillation	Partial condensation process	98–99	75–80	55 000	18–100	Mature technique	High capital and operating costs
						High CO purity	Unable to separate CO and N_2_ due to the similar boiling points
	Liquid methane wash process	99	99	34 000	300–600		Pretreatment removal of H_2_O and CO_2_ necessary

Absorption	COSORB	99	99	270–5500	1446 (cooling/reboiler)	High purity and recovery of CO product	The CuAlCl_4_ complex can easily be poisoned
					392 (kW electrical power/tonne CO)	Can separate CO/N_2_	The solvent degradation and the precipitation of solids need to be considered.
	Copure^SM^	99	99	Not known	Not known		Environmental and safety issues

Membrane separation	Prism	97	89	Not known	Not known	The process can be operated in the presence of H_2_O and CO_2_	Poor stability of the material
						Can achieve desired H_2_/CO syngas ratio	Multistage separation is often needed in order to achieve high purity and recovery
	Cellulose acetate	98	88	2200	Not known	Reduce the environmental impact due to the absence of chemical systems	Cannot separate CO/N_2_

Adsorption	COPISA	98	45	450	Not known	Easy operation	A pretreatment is needed to remove H_2_O and sulfur components.
	Activated Al with impregnated C & Cu compound	>99	>80	≥150	Not known	Low cost and relatively energy-efficient for the separation of CO/N_2_	Only for high CO concentration feeds
	PU-1	>99	>85	1700–20 000	340	High CO product purity, with modest to relatively high recovery	

Flue gas streams often also contain large amounts of CH_4_ and N_2_, which are hard to separate from CO based on conventional separation principles like boiling point (cryogenic distillation) and kinetic diameter (membrane separation). Design of such N_2_/CO separation unit results in expensive and energy intensive solutions to reach required purities of 98–99%. This explains the preference for reactive separation in both absorption and adsorption processes exploiting the affinity differences of CO and N_2_ towards binding in d-metal complexes to selectively extract CO. The exception being the COPISA process based on a sodium-type-mordenite. Most likely the higher quadrupole moment and polarisability of CO compared to those of N_2_ lead to a stronger interaction with sodium. Yet, the CO/N_2_ selectivity of 2.5–3 is only modest, hence, the desired high purity (98%) can only be achieved *via* a lower recovery (*ca.* 45%). The other commercialised absorption and adsorption processes are based on cuprous complexes. The Cu(i)–CO π-complexation bonds are stronger than the van der Waals interactions in the COPISA process. This means that high purity (99%) can be achieved with high recovery (80–99%). So, chemisorption based processes are needed to achieve high purity at high yield, even when the CO concentrations in the feed are high. The formed Cu(i)–CO bonds are still weak enough to be broken by traditional engineering means such as decreasing pressure and/or increasing temperature. This reliance on heating for cyclic operation, *i.e.*, switching between ab/adsorption and desorption operation modes is a disadvantage of the ab/adsorption process, especially as heating and cooling stages are typically more time-consuming compared to changes in pressure. Moreover, the low solubility of the metal complexes in the hydrocarbon solvents limits the uptake capacity, resulting in energy intensive heating and cooling stages of the process (1446 kW h needed per tonne CO). Additionally, solvent degradation may cause environmental issues.

Overall, relatively adequate solutions seem to exist for the separation of CO and H_2_. They can be lean in energy use, *e.g.*, cryogenic distillation based on the partial condensation process, and probably – though no information is present in literature – membrane separation. The latter has the additional advantage that no preconditioning to remove H_2_O and CO_2_ is needed.

For the separation of CO and N_2_, the options for high purity solutions are far more limited. Absorption processes can yield high purity with high recovery, but at a high energy cost and with environmental concerns related to the use of solvents. Adsorption processes, on the other hand, are more energy efficient. A techno-economical analysis comparing the COSORB absorption process and the Kobe steel adsorptive Linze-Donawitz gas (LDG) treatment revealed the favourable economic performance of the adsorption process.^57^ The environmental concerns are lower, as no solvents are used. However, the CO recovery is lower (45–85%) and the process is only sensible for separating flue gas streams with a high CO content. A real impact can be made by a process for flue gas separation of CO from N_2_ with high recovery and purity, even if the stream only contains a few percent of CO. Especially if such a process would not require the need a pretreatment to remove CO_2_ and H_2_O.

## 3 Current development in CO separation

The vast plethora of new materials identified the last decade as capable of separation CO concerns adsorbents, which is perhaps not surprising considering the advantages of CO separation *via* adsorption (simple equipment, process operation at moderate conditions and low energy requirements) and the limitations of current separation technologies (Section 2.4). It has been reported that many solid materials, such as activated carbons, zeolites, and metal–organic frameworks (MOFs), are capable of adsorbing CO to a certain extent. Therefore, we provide a comprehensive overview of these adsorbents (Section 3.2), pointing out essential further development of suitable CO adsorbents. We note a lack of criteria on the evaluation of adsorption performance, which are, however, critical to assess the large-scale viability of the adsorption process. Therefore, in Section 3.1, evaluation criteria for the CO adsorption performance are suggested. Using these criteria, the performance of each type of adsorbent from the last decade under different adsorption conditions (*e.g.*, concentration, pressure, temperature, *etc.*), and the relation with structure is discussed. Meanwhile, the benefits and limitations of each type of adsorbent will be highlighted. Most importantly, we will summarise the key factors that control the CO adsorption performance of different materials and point out the most promising application. Also, the literature on emerging absorbents and membranes for CO separation is discussed (Section 3.3), and their performance contrasted with that of the adsorbents. This evaluation of materials provides a wish list of desirable properties for future materials designed for this specific application.

### 3.1 Key factors that control the adsorption performance

#### 3.1.1 Ideal adsorbent for CO adsorption

The advent of material science and approaches borrowing from inorganic–organic chemistry opened new opportunities to develop novel adsorbents. Over the past decade, the research conducted to identify solid adsorbents for carbon monoxide (CO) capture has rapidly expanded. Several solid adsorbent materials, such as activated carbon, zeolites, silica gel, mesoporous alumina, and metal–organic frameworks, have exhibited favourable properties for CO adsorption, each with its own set of thermodynamic and structural characteristics, such as adsorption/desorption isotherms, number of active sites per area, surface area per mass of adsorbent, pore structure and, porosity.^12^ However, identifying the ideal adsorbent for CO in an industrial process is not a straightforward task as it requires optimisation of thermodynamic and structural characteristics along with relevant transport phenomena while considering process requirements.^51,58^ The thermodynamic properties of an adsorbent such as adsorption/desorption isotherms and selectivity are characterised and correlated to nano/mesoscopic structure characterisation on lab scale.^59^ These thermodynamic and structural characterics enable process engineers to estimate the extent of achievable separation and the amount of CO adsorbed per weight of CO absorbent under ideal conditions. As industrial operations occur under dynamic conditions, determination of breakthrough profiles under conditions mimicking cyclic processes are essential in providing information of coupled mass and heat transport phenomena. In literature, very few materials are tested with benchtop pre-pilot experiments, even fewer reach pilot scale where harsh process conditions can quickly alter adsorption performance. Any proposed adsorbent for CO separation should be tested in humid conditions as well as in the presence of N_2_, due to their ubiquitous nature and the challenges these compounds cause in current industrial processes (Section 2.4). Moreover, practical considerations such as adsorbent stability under process conditions, especially regeneration, resistance against attrition, materials cost and availability are important. Together with process design specifications and heat integration possibilities these collectively shape what an ideal adsorbent is for a given process. These considerations are discussed in subsequent sections.

#### 3.1.2 Thermodynamics and selectivity

In CO adsorption, the nature of interaction with an adsorbent is the key factor directly determining the thermodynamics, the adsorption/desorption isotherm and, hence, the maximum achievable separation performance. Depending on the strength of this interaction, the adsorption process is classified as physical or chemical in nature, the later possessing stronger interactions (see Section 2.2). Assessment of thermodynamics of a new CO adsorbent, such as standard Gibbs free-energy change (Δ*G*°) and the enthalpy change (Δ*H*°), provides information about the nature of adsorbent–adsorbate interactions^60–62^ and the kind of sorption, *i.e.*, the presence of various forces, including van der Waals, hydrophobic/hydrophilic interactions, hydrogen bonds, ligand exchange and dipole–dipole interactions. In general, the magnitude of −Δ*H*° for absolute physical adsorption is <20 kJ mol^−1^, while it is within the range of 80–200 kJ mol^−1^ for chemisorption.^63,64^ Notably, an exothermic adsorption process (Δ*H*° < 0), releases energy in the form of heat to its surroundings, and includes either physisorption, chemisorption, or a mixture of both (comprehensive adsorption). By contrast, an endothermic process (Δ*H*° > 0), in which heat is absorbed from the surroundings, is unequivocally attributable to chemisorption.^65–68^ For any proposed material CO adsorption should happen spontaneously, *i.e.*, Δ*G*° < 0. When Δ*G*° > 0, the process will be neither feasible nor spontaneous.^69,70^

In addition to desirable thermodynamics, selectivity has to be a key feature of CO adsorbents, particularly for complex gas streams commonly encountered in industrial practice. In physical separation processes, the difference between CO and N_2_ is small causing problems separating the two as shown in Section 2.4.1, 2.4.3, and 2.4.4. Physisorption suffers from a similar problem. Due to the physical similarities between CO and N_2_, N_2_ tends to occupy the same physisorption sites, strongly reducing both the adsorption capacity of CO and selectivity towards CO when N_2_ is present. Chemisorption can solve the issue of separating CO and N_2_ due to its increased selectivity towards CO, but most CO sources also have a considerable humidity. Water will also compete for adsorption sites and may cause issues in stability of the adsorbents. Therefore, either water must be removed prior to adsorption process with a separate dehumidification step, as outlined in Section 2.4, or robust adsorbents capable of operating under humid conditions should be developed.^58^

#### 3.1.3 Structure, adsorption capacity, and mechanical properties

As adsorption is a process that takes place on the surface of the adsorbent, structural characteristics such as available surface area, porosity, and the number of sorption sites per adsorbent mass determines the maximum amount of CO that can be adsorbed per unit mass, *i.e.*, the capacity.^71^ In other words, the adsorbent capacity is the characteristic collectively dictated by thermodynamics and texture. As a general rule, a larger specific surface area of an absorbent results in more sorption sites and a higher capacity to adsorb another material.^72^ Moreover, the pore size, shape, and size distribution, *i.e.*, texture, dictates the gas transport properties, so the dynamic adsorption rate.^65^

Pores in absorbents are classified in three categories according to IUPAC.^73^ Macropores with diameter, *d*_pore_, more than 50 nm (*d*_pore_ > 50 nm) and mesopores (2 nm ≤ *d*_pore_ ≤ 50 nm) are primarily relevant for mass transport into the interior of the adsorbent particles; whereas, micropores (*d*_pore_ < 2 nm) constitute the largest portion of the internal surface of an adsorbent and contribute most to total pore volume and capacity.^71^ The molecules adsorbed are transported through the macropores into the mesopores and finally into the micropores. In the micropores the attractive forces are dominant and most of the gaseous adsorbates are adsorbed within that region. Industrially implemented processes for CO separation, such as COPISA (Section 2.4.4.1), use a combination of carbon black particles with a broad pore size distribution along with zeolite with only micropores to separate CO from a mixture of CO, CO_2_ and N_2_. The most common problem related to pore size is the blockage of pores during operation. These blockages originate from impurities limiting the effective capture of CO.^65^ Thus, careful consideration and balance of all the porous characteristics are crucial for determining the ideal adsorbent to capture CO under a given set of conditions.

From a structural perspective, adsorbents are divided into two broad classes: homogeneous and composite adsorbents. Homogeneous adsorbents comprise a similar pore network throughout the particle, whereas composite adsorbents are formed by aggregation of small microporous microparticles. As a result, composite particles have a well-defined bimodal character with micropores within the microparticles connected through macropores within the pellet.^72^ The commercialised COPISA process uses a homogeneous zeolite adsorbent while the Kobe steel process uses a heterogeneous adsorbent with activated alumina as the carrier and carbon impregnated with CuCl as the active material.

An ideal CO adsorbent should withstand packed bed weight or resist attrition during moving bed operation. This requirement is often at odds with multiscale porous structures promoting the mass transport of the adsorbates while offering a large surface area. As a rule of thumb, the more porous the structure is, the more fragile it becomes. Resistance to attrition is particularly relevant for moving and circulating bed adsorption designs.^72^

#### 3.1.4 Transport phenomena and scaling

Testing of novel adsorbents in bench-top dynamic breakthrough experiments helps in gaining information about mass transport properties (Section 3.1.1). Particularly, breakthrough time and width are used as a first estimate in scale up calculations. However, these lab-scale experiments cannot entirely replace pilot scale or industrial scale experiments alone. The coupled heat, mass, and momentum transfer phenomena and their corresponding coefficients do not simply scale linearly as the process is scaled up from bench-top to industrial scale. The observed adsorption kinetics in an industrial process is collectively dictated by the mass transfer rate of the species from bulk to the adsorbent active site. In general, the overall rate of physical adsorption is controlled by limitations in mass and heat transport rather than by the actual rate of equilibrium adsorption/desorption inside the adsorbent pores. Chemisorption often introduces slower desorption kinetics due to the higher binding energies. Consequently, physisorption processes are more commonly encountered in industrial CO adsorption when dealing with pre-treated feed streams with high-capacity separations as in the case of the COPISA process (Section 2.4.4.1). Chemisorption, on the other hand, is preferred to reach higher selectivity as in the case of the Kobe Steel Ltd process (Section 2.4.4.2).

In industrial scale processes not only mass transport influences adsorption process design, heat transport can also be a critical factor in a cyclic TSA or (V)PSA. The largest drawback of industrial scale TSA implementations is the large cycle times needed if gas–solid contacting devices are restricted in fixed bed designs. Heating and cooling times in packed bed or pellets are in the order of hours due to poor heat transport in these systems. Consequently, (V)PSA is preferred in fixed bed designs over TSA in industrial systems although thermal effects also interfere strongly due to the exothermicity of adsorption. As a solution to reduce the long heating time in meter sized adsorption processes, circulating fluidised beds or moving beds have been proposed.^58^ Despite the fact that circulating fluidised beds or moving beds are more difficult to operate and require higher capital investment costs, they offer a solution to long heating times in fixed bed TSA processes.

#### 3.1.5 Regeneration and stability

The adsorbents used in CO separations must also be regenerated after extended use. Therefore, this regeneration process must be conducted efficiently and without damage to the adsorbents mechanical and adsorptive properties. The performance of regeneration step is dictated by the nature of adsorbent–CO interactions as well as mass transfer.^74^ The regeneration step is also important in the context of the economic and environmental aspects in the process design. In the current era of sustainable development and circularity, it is imperative that designed adsorbents permit the effective regeneration, recovery, and re-use of the adsorbate, a topic often overlooked in CO adsorption literature.^75,76^ One of the environmental issues associated with adsorption processes is disposal of the adsorbent at the end of its life cycle. Regeneration can reduce the requirement for new adsorbent materials while simultaneously alleviating the problem of adsorbent waste. Furthermore, industrial adsorption processes are usually cyclic, during which the adsorption and regeneration steps of the adsorbent material change periodically; regeneration is often critical, since it determines the length of the cycle and the energy efficiency.^77^ Although all the commercial CO adsorption processes described in Section 2.4.4 are cyclic, no information is available on the recycling of the adsorbents. Poisoning of the adsorbents by impurities such as SO_*x*_ and NO_*x*_ along with attrition due to thermal and mechanical stresses are the most common issues in regeneration.^58^

#### 3.1.6 Cost and accessibility

One of the most critical factors influencing the industrial scale implementation of any adsorption process is the costs involved. Especially for industrial purposes, this is the key factor in the comparison and selection of adsorbents. An adsorbent can be considered low cost and accessible if it requires minimal processing and is made of abundantly accessible materials. The cost of the entire process is dictated by capital investment (CAPEX) that is influenced by the design choices shaped by adsorbent and operational costs (OPEX) that include the cost of adsorbents that need to be regularly replaced due to attrition and irreversible adsorption of impurities. The cost of porous adsorbent material will be directly proportional to the quantity required in the process, which in return will be determined by their effectiveness in capturing CO. Therefore, there is a need to develop low-cost and easily available materials that can be used on a large scale in an economically beneficial way.^78^ For CO adsorption, commercial processes such as COPISA use a multi-step process with abundant and low-cost activated carbon for CO_2_ and a zeolite based adsorbent (sodium-type-mordenite) as CO adsorbent, whereas activated alumina porous materials with a copper impregnated carbon is used at Kobe steel, Japan, for extraction of CO from metallurgic flue gasses. These industrial implementations underline the importance of using low-cost and abundant materials for any novel process.

In addition to the direct costs of adsorbent, the heat required for switching between adsorption and desorption also known as swing operation in TSA should be considered as operational costs, while in PSA this is represented by compression costs. The preferred heat of desorption is intimately dictated by the available heat streams available for heat integration at site. For instance, for adsorption from post combustion flue gas, the isotherm of the adsorption must show a strong change in uptake within the temperature range 30–150 °C. This is the range of temperatures at which heat is available in the power plant (*e.g.*, low pressure turbine, flue gas waste heat, CO_2_ compression after cooler heat, *etc.*) and that can be used to contact the adsorbent either directly or indirectly. In addition to direct heating, alternative heating strategies such as indirect heating in fixed beds^79^ and even electrical heating systems^80^ in structured packings have been proposed.

#### 3.1.7 Criteria for the evaluation of adsorption performance

A viable industrially implementable CO adsorption process should feature a high adsorption capacity with favourable CO adsorption/desorption isotherms allowing significant changes within a reasonable temperature or pressure range, as well as considerable operational and mechanical sorbent stability over a broad range of process conditions along with low cost. Some of the desired properties are (i) high and sustained CO adsorption capacity even in the presence of impurities such as SO_*x*_, NO_*x*_ and occasionally HCl, which may be present in the flue gas; (ii) high selectivity for CO and preferably not adsorbing nitrogen and water; (iii) rapid adsorption kinetics and low energy requirement for swing operation between adsorption and desorption; (iv) high resistance to attrition, particularly for circulating and moving bed process implementations; (v) and low corrosion sensitivity for the feed stream. Considering the commercially available processes any novel adsorbent with high adsorption capacity should comply with these criteria. In the next section we will consider different materials and evaluate their performance on these aspects.

### 3.2 Different types of solid adsorbents for CO adsorption

Since the performance of the adsorption separation technology highly depends on the adsorbent material, it is of importance to carefully choose or design it. It has been reported that many solid materials, such as activated carbons, zeolites, and metal–organic frameworks (MOFs), *etc.*, are capable of adsorbing CO to a certain extent. Hence, it is necessary to provide a comprehensive guide to the essential development of suitable CO adsorbents at this stage. In this section, we will review and analyze the performance of each type of adsorbents from the last decade under different adsorption conditions (*e.g.*, concentration, pressure, temperature, *etc.*), as well as the relationships between their structures and the application performances. Meanwhile, the benefits and limitations of each type of adsorbent will be discussed. Most importantly, we will summarise the key factors that control the CO adsorption performance of different materials and point out the most promising technique. An insight into desirable properties to include in future materials designed for this targeted application will be provided.

#### 3.2.1 Activated carbon and other carbon-based porous solids

Activated carbon (AC) is one of the most widely used adsorbents for gas separation. It is composed of “pores” consisting of different sizes and shapes, which may vary over a large range. Different precursors and the preparation methods determine the internal pore structure and surface characteristics of the AC. It may have a BET surface area ranging from 500 m^2^ g^−1^ to 1500 m^2^ g^−1^, which is associated with functional groups on the pore surface. Meanwhile, the total pore volumes can change between 0.3–0.8 cm^3^ g^−1^. These large differences have a significant impact on the adsorptive characteristics of the components of a gas mixture to be separated.^81^

Lopes *et al.*^82^ studied the ability of activated carbon for the separation of gas mixtures from steam methane reforming. It is reported that the adsorption capacity of the gases follows the order as CO_2_ > CH_4_ > CO > N_2_ > H_2_ with a CO adsorption capacity of 0.4 mmol g^−1^ and CO/N_2_ selectivity of 1.3 at 1 bar. Therefore, it is difficult to use AC to selectively separate CO from those gas mixtures because of the lower adsorption capacity and selectivity. This is evidenced by the small differences in isosteric heats of adsorption between CO_2_ (−29.1 kJ mol^−1^), CH_4_ (−22.7 kJ mol^−1^), N_2_ (−16.3 kJ mol^−1^) and CO (−22.6 kJ mol^−1^). Additionally, all isosteric heats of adsorption reported are in the range that can be classified as physisorption, which could perhaps explain the unselective nature of activated carbon adsorbents for these gases. Meanwhile, activated carbon is non-polar or slightly polar with a high surface area, which gives it the advantage of adsorbing non-polar or weakly polar molecules. To use it for CO adsorption/separation purpose, active metal sites (*e.g.*, Cu(i)) are introduced on the surface of the activated carbon with impregnation method.

The formation of strong π-complexation bond between Cu(i) and CO can be a benefit for CO adsorption process showing high selectivity and capacity. The bond will further be broken by raising the temperature or decreasing the pressure of the system. The adsorption/desorption of CO on activated carbon impregnated with metal halides (AgCl, CuCl, CuBr, CuI, FeCl_2_, FeCl_3_, NiCl_2_, PdCl_2_ and ZnCl_2_) was measured *via* fixed bed runs (measured at 1 bar, 0.05 mol% CO, 323 K). Only the copper halides and PdCl_2_ showed a higher CO uptake in comparison to unimpregnated carbon, with PdCl_2_ 20 times and CuCl 8 times that of the unimpregnated activated carbon, and the uptake decrease as CuCl > CuBr > CuI. For the Cu halides, all CO could be desorbed without heating, while for PdCl_2_ it was necessary to heat to 423 K.^83^ Inspired by this, many works have been done to synthesise adsorbents with monolayer dispersed Cu(i) on the surface of AC to adsorb CO molecules. CuCl is usually chosen instead of bare Cu(i) due to its weakened Cu–CO binding interaction, as the bare Cu(i) does not allow for facile desorption even at pressures as low as 100 Pa at temperatures around 100 °C.^84,85^

The metal sites can be formed on the pore surfaces by direct dispersion and impregnation of CuCl. Hirai *et al.*^84^ used various solvents, such as water, concentrated hydrochloric acid solution, acetonitrile, and toluene, to disperse CuCl and activated carbon as to impregnate the CuCl into the activated carbon. After the impregnation the CuCl/activated carbon was dried under an inert atmosphere creating a uniform distribution of CuCl throughout the activated carbon. The usage of the various solvents leads to a range of CuCl loading (mmol g^−1^ AC): 0.84 (water), 1.41 (3 M HCl), 0.92 (acetonitrile), and 0.20 (toluene). These loadings in turn lead to a CO adsorption capacity (mmol g^−1^) of 0.56 (water), 1.24 (3 M HCl), 0.61 (acetonitrile), and 0.13 (toluene) in which the highest CO/CuCl ratio of 0.88 is found from the preparation in 3 M HCl. In a further study, in which the amount of CuCl is changed for a set amount of 3 M HCl and activated carbon, it is observed that higher loading of CuCl in the activated carbon significantly reduces the BET area by up to 50% at a CuCl loading of 4.1 mmol g^−1^ AC, which in turn leads to a lower CO/CuCl ratio which decreases strongly after a CuCl loading of 2.5 mmol g^−1^ AC.

Another method that was used instead of the drying under an inert atmosphere is the calcination of the activated carbon after impregnation as shown by Huang *et al.*^86^ CuCl and activated carbon were dispersed in hexane, after which the activated carbon was calcined under argon at 623 K. A maximum loading of CuCl in the activated carbon of 16.16 mmol g^−1^ was observed, while the optimal loading is found to be 12.12 mmol g^−1^ with a maximum observed CO adsorption of 1.98 mmol g^−1^ at 0.9 *P*/*P*_0_ and a CO/N_2_ selectivity of 100–450 at 0–10 kPa. The maximum adsorption of 1.98 mmol g^−1^ means that only 18.6% of the CuCl is utilised at 0.9 *P*/*P*_0_ under optimal loading, which is a significant decrease compared to the work of Hirai *et al.* This underutilisation of impregnated CuCl is caused by reduction of Cu(i) to Cu(0), attributable to carbon acting as a reducing agent in the calcination, clustering of CuCl which is caused by higher loading of CuCl, and oxidation of Cu(i) to Cu(ii), which probably occurs during the initial mixing step.

In both the method of Hirai *et al.*^84^ and Huang *et al.*,^86^ preparation of the CuCl needs to (partially) be done in a dry, inert atmosphere as to prevent oxidation of the Cu(i) rendering the material useless. The usage of CuCl is, therefore, not suitable for larger scale production. A more suitable alternative to CuCl is the usage of stable CuCl_2_ during impregnation which can then later be reduced to CuCl.

Gao *et al.*^87^ proposed such a method in which they ground CuCl_2_·H_2_O and activated carbon together, after which the CuCl_2_ was reduced at 453 K under nitrogen flow to CuCl. CuCl loadings of 4.0, 5.0, 6.0, 7.0, and 8.0 mmol g^−1^ AC were produced with an adsorption capacity of 2.44, 2.59, 2.77, 2.95, and 2.95 mmol g^−1^ at 1 bar, 303 K. Optimal CuCl loading was, therefore, determined to be 7.0 mmol g^−1^ AC, as increasing the CuCl loading did not increase the capacity any further. Selectivity compared to other common gases in industrial streams such as CO_2_, CH_4_, and N_2_ is high, with selectivity values for CO/CO_2_, CO/CH_4_, and CO/N_2_ of 6.28, 16.39, and 42.14, respectively. The material is also stable after multiple uses with a reversible CO adsorption capacity of 2.53 mmol g^−1^ at 1 bar and 303 K which remains stable during 6 cycles of ad- and desorption.

In order to utilise this adsorbent in a real PSA process Gao *et al.*^88^ later reported a simulation of a five-bed and 7-step VPSA process with Aspen software. After optimising the operating conditions, a high recovery of 92.9% with 98.1 vol% purity of CO can be achieved from the syngas (32.3 vol% CO, 1.0 vol% CO_2_, 2.4 vol% CH_4_, 18.3 vol% N_2_, 46.0 vol% H_2_).

Ma *et al.*^89^ reported a solution impregnation method to disperse Cu metal salts on activated carbon. The activated carbon was added to an aqueous solution containing CuCl_2_, CuCl_2_ and Cu(CH_3_COO)_2_, or CuCl_2_ and Cu(HCOO)_2_. After removing the water and activating the solid at high temperature under a reducing atmosphere, the Cu-based adsorbents were obtained. The most efficient adsorption is found at a loading of Cu(i)-ions of 4 mmol g^−1^ AC with an adsorption of 0.98 mol CO/mol Cu(i) using CuCl/Cu(HCOO)_2_ in a 1 : 1 ratio. It is reported that only when the mixture of CuCl_2_ and Cu(HCOO)_2_ is used as starting material, the Cu(ii) salt supported on AC can be reduced completely to highly dispersed CuCl after activation resulting in an adsorption capacity of 2.28 mmol g^−1^ at a loading of 4 mmol Cu(i)/g AC which is equal to that obtained by the dry grinding method of CuCl and activated carbon. Meanwhile, the CuCl_2_/Cu(CH_3_COO)_2_, 1 : 1 ratio, results in a CO adsorption capacity of 1.67 mmol g^−1^ at 4 mmol Cu(i)/g AC. Although the introduction of copper causes a decrease in BET surface area and pore volume in the activated carbon, its CO adsorption capacity increases, meaning that the CO adsorbs onto Cu(i) ions. The stronger interaction between Cu(i) and CO also leads to a relatively high CO adsorption selectivity over CO/CO_2_, CO/CH_4_ and CO/N_2_ of 3.2, 35, and 9, respectively, at 1 bar and 298 K. The adsorbent shows a good adsorption–desorption reversibility thanks to the average adsorption heat of CO (25 kJ mol^−1^). However, the CO adsorption capacity does decrease rapidly when the adsorbent is exposed to air for several hours.

Relvas *et al.*^90^ used a wet impregnation method, first reported by Golden *et al.*,^91^ which adds a dispersing agent to the Cu salt solution to make the adsorbent for a PSA system which can be used to produce pure H_2_ gas. First, they pretreated the AC at high temperature under air to make its surface more hydrophilic, increasing the distribution of the Cu. After letting the AC cool down slightly, the AC was placed in an aqueous CuCl_2_·2H_2_O solution in which ammonium citrate was added as the dispersion agent. The AC was then activated under a reducing atmosphere at elevated temperature. 5 different loading were tested and compared: 0, 0.5, 2, 3.5, 5 mmol Cu per g adsorbent. The highest CO adsorption capacity, CO/CO_2_ and CO/H_2_ selectivity all belong to the 5 mmol Cu per g adsorbent with values of 2.01 mmol g^−1^, 4.5, and 200, respectively. Taking into account the need to remove both CO and CO_2_ they used the 2 mmol Cu per g of adsorbent system to produce a H_2_ stream containing only 0.17 ppm CO from an input stream containg 1% CO.

Though the Cu(i) adsorbents can be prepared by impregnating Cu(ii) salts into activated carbon and then reducing Cu(ii) to Cu(i) using a reducing agent, it is sometimes difficult to control the reduction degree, as Cu(ii) is easily overreduced to Cu(0). Based on this consideration, Xue *et al.*^92^ reported a solid-state auto reduction–dispersion method with Cu(ii) metal salts to develop Cu(i) dispersed AC adsorbent. The activated carbon was mixed with CuCl_2_ and Cu(HCOO)_2_ salts in solid state and the Cu(ii) salts were transformed into CuCl after activation at 533 K under vacuum. It is found that the CO adsorption capacity increases with the increasing of CuCl loading from 0 to 4 mmol g^−1^. The highest CO adsorption capacity of 1.85 mmol g^−1^ is obtained when CuCl loading in the activated carbon is 4 mmol g^−1^ with selectivities towards CO/CO_2_, CO/CH_4_, and CO/N_2_ of 2.6, 8.0, and 34, respectively, at 1 bar. This adsorption capacity remains constant during six adsorption/desorption cycles. Increasing the copper loading above 4 mmol g^−1^ AC results in a decrease of the surface area of AC and an agglomeration of Cu(i) on the AC surface. As a result, the active sites decrease as well as the amount of adsorbed CO.

Thanks to the π-complexation bond formed between Cu(i) and CO, which is stronger than the van der Waals force, the Cu(i) doped AC adsorbents show higher adsorption capacity and higher selectivity of CO than AC. Moreover, these bonds can be broken by raising the temperature and/or decreasing the pressure, such as shown in the Aspen simulations done by Gao *et al.*^88^ in which pressure was changed between 6 bar and 0.14 bar, while keeping the temperature at 303 K for their VPSA, showing that these adsorbents always possess good adsorption and desorption reversibility. However, the disadvantage is that the stability of Cu(i) is very poor in air and the adsorbents need to be protected by a dry, inert atmosphere.

A recent study by Kwon *et al.*^93^ using petroleum based and sulfur doped activated carbon supported Ni showed an exceptionally high CO adsorption capacity in a TSA setup (6.56 mmol g^−1^ at standard temperature and pressure) owing to the strong interaction between well-dispersed Ni(0) atoms and CO. The presence of sulfur enhances both the adsorption capacities and the desorption characteristics of the adsorbent. Unfortunately, no IAST (Ideal Adsorbed Solution Theory) selectivities are reported for comparison and the Ni loading was quite high (10 wt%), but a reversible uptake of 3.57 mmol g^−1^ could be reproduced for 10 cycles with an overall loss in activity of 4.8%.

Other than Cu and Ni, an activated carbon impregnated with SnO_2_ was employed in a pressure swing adsorption system as an adsorbent to remove CO from H_2_/CO mixture. The CO adsorption capacity of the SnO_2_/AC is 1.43 mmol g^−1^. The species that are responsible for the improvement of the adsorption ability is SnO_2_. However, the adsorption capacity of CO with SnO_2_ is much lower compared to Cu(i), which is due to the weak physisorption interactions between CO and SnO_2_. The mechanism is that CO reacts with O^−^ on the AC-SnO_2_ surface at high pressure and ambient temperature and forms a CO-(O-)SnO_2_-AC complex. The simulation results show that the adsorbent has a CO recovery and purity of 99.99% and 57.48% at a cyclic time of 600s in PSA process operating between 1 and 10 atmospheres.^94^

Yoon *et al.*^95^ proposed a porous organic polymer with chelating N-sites for the embedding of CuCl. The polymer, SNW-1, was synthesised solvothermally by combining melamine and terephthaldehyde in dimethyl sulfoxide at 180 °C in a Teflon liner for 10 hours. After purification of the polymer, SNW-1 was added to a CuCl in acetonitrile solution and stired for 72 hours at 25 °C under a reflux condenser, yielding *n*Cu(i)@SNW-1 with n depending on the amount of mg CuCl/mg SNW-1. This resulted in an increase in dynamic CO capacity (0.1 to 1 bar) from 0.31 mmol g^−1^ with bare SNW-1 up to 0.61 mmol g^−1^ at 1.3Cu(i)@SNW-1. The AIST CO/CO_2_ selectivity similarly increases from 0.10 on bare SNW-1 to 23.3 on 1.5Cu(i)@SNW-1. Similar materials based on graphitic carbon nitride were also reported as CO-sensor materials,^96,97^ however, only their strong interaction with CO is noted.

#### 3.2.2 Zeolites

Another type of porous materials with an extensive library of high surface area structures that could be used as adsorbents for capturing CO is zeolites. Zeolites are crystalline aluminosilicate structures originally found in nature, but nowadays mass produced to be used as commercial adsorbents. They generally consist of structured cages, which can contain a large number of guest molecules. Negative charges caused by exchange of Si^4+^ by Al^3+^ are balanced with cations (*e.g.*, Na^+^, K^+^, Ag^+^) and the size of the window apertures, which can range from 3 Å to 10 Å, depends on the zeolite crystal structure, as well as the type and number of cations. These cations are loosely bound to the structure and can be exchanged with other cations by contacting the zeolite to a cation-containing solution. It is found that the Si/Al ratio, pore size, chemical composition, and the location of extra framework cations in zeolites greatly influences the adsorption capacity and gas adsorption selectivity.

Zeolites have been investigated as potential materials for CO adsorption and separation. As the Al and Si atoms are present at the centre of tetrahedral units, thus not directly accessible by the gas molecules, the interaction of the gas molecules with the zeolite surface are mainly with the lattice oxygen atoms and accessible extra framework cations. For ZSM-5^98^ with Si/Al ratio's varying from 25 to 900, and Na^+^ as countercation. The CO adsorption capacity and CO/N_2_ selectivity increases as the Si/Al ratio decreases due to an increase in the sodium cations per unit cell. For example, at 1 bar and 303 K, the CO/N_2_ selectivity is 1.8 and the CO capacity is 0.93 mmol g^−1^ for Si/Al = 25, while only being 1.4 and 0.27 mmol g^−1^ for Si/Al > 400. Thus, the electrostatic interaction between the countercations and the CO molecules is important. The quadrupole moments, the polarisability, and the dipole moment of the gas molecules determine the magnitude of these electrostatic interactions. A stronger interaction between Na^+^ and CO compared to N_2_ is observed because of the higher quadrupole moment and polarisability of CO compared to that of N_2_.^98^ While ZSM-5 is a medium pore zeolite, it is observed that for narrow-pore zeolites (*e.g.*, Linde Type A (LTA)) the pore size can become limiting for diffusion of CO. In zeolite 3A specifically (LTA with K^+^ as countercations) the effective pore aperture is only 3 Å, while the kinetic diameter of CO is 3.69 Å. Indeed it has been shown that CO can only absorb on the outer surface of zeolite 3A, in contrast to LTA-zeolites with a slighlty larger pore size like zeolite 4A (Na^+^ cations) and zeolite 5A (Na^+^ and Ca_2_^+^ cations).^99^ In zeolite 5A the adsorption capacity of CO at 298 K and 1 bar is 1.2 mmol g^−1^.^100^

By far the most research has been done on large pore zeolites with a faujasite-type structure (pore aperture ≈8 Å), like zeolite X and Y, with zeolite X usually having a Si/Al ratio of 1–1.5 and zeolite Y having a Si/Al ratio above that.^101^

Pillai *et al.*^102^ studied the sorption of CO in different alkali metal ions exchanged Zeolite-X materials (Si/Al = 1.18) in which it is observed that both the equilibrium adsorption capacity of CO and its adsorption enthalpy decreases as the cation element goes down the period table: LiX > NaX > KX> RbX > CsX. This is due to the increase of the cation radius from Li^+^ to Cs^+^, which again leads to a decrease of the electrostatic interactions between CO and the metal ions. As such, the LiX shows a higher CO adsorption capacity than CsX. The selectivity of CO/N_2_ is around 2 for all alkali metal ion exchanges zeolite X materials.

Sethia *et al.*^103^ exchanged the Na^+^ in Zeolite X with alkaline earth metal ions (Mg^2+^, Ca^2+^, Sr^2+^ and Ba^2+^). Apart from MgX, the CO adsorption capacity of alkaline earth metal ion exchanged Zeolite X is higher than those exchanged with alkali metal ions. The adsorption enthalpy again (excluding Mg^2+^) decreases with increase of the cation radius: CaX > SrX > BaX. Thus the electrostatic interaction between the quadrupole and dipole moment of CO and the earth alkaline ions is enhanced by the increase of charge density of the cation. MgX, the most charge dense ion, has a distincly lower CO adsorption capacity and adsorption enthalpy. This is due to its very small ionic radius: during activation Mg^2+^ ions migrate from the supercages to the smaller sodalite cages where they are not able to effectively interact with the CO molecules. Among the alkaline earth metal ions Sr^2+^-exchanged Zeolite X shows the highest CO capacity of 1.90 mmol g^−1^ at 303 K and 1 atm.

Ion-exchanged zeolite Y, which has the same crystalline structure as zeolite X, but a higher Si/Al ratio and, thus, fewer counter cations, was also studied both in the proton exchanged form with Si/Al = 5^104^ and in the Na^+^ form with Si/Al = 2.4.^105^ The former shows a CO capacity of 0.21 mmol g^−1^ (303 K, 1 bar) and the latter 0.48 mmol g^−1^ (293 K, 0.6 bar). The lower performance compared to zeolite X is probably due to the presence of relatively few counter cations.

The above research shows that although the effect of the bound alkali and earth alkaline cation can improve the CO adsorption capacity of zeolites to some extent due to the electrostatic interactions, the overall capacity of ion-exchanged zeolite-based adsorbents is relatively modest with a maximum value of 1.90 mmol g^−1^ observed for Sr-exchanged zeolite X.

Therefore, more effort was put into using zeolites as high surface area supports for transition metal salts, especially Cu(i)-salts, for selective capture of CO.^106^ As mentioned in, the commercialised CO adsorbent PU-1 is made by heating a mixture of CuCl and a zeolite at a suitable temperature to disperse the CuCl onto the surface of the zeolite (the type of zeolite is not further specified). The researchers prepared a series of adsorbents by mixing CuCl with different porous supports (γ-Al_2_O_3_, zeolite 4A, 13X, NaY, Cu(i)Y) and heating the mixtures at 350 °C for four hours under nitrogen.^105^ It is found that the CO adsorption capacity of all adsorbents increases after doping with CuCl. Among them, the CuCl/NaY (3.66 mmol g^−1^) and CuCl/Cu(i)Y (4.05 mmol g^−1^) have the highest adsorption capacity of CO at 293 K and 60 kPa, and also simply the highest optimal loading of CuCl in the support. The CuCl optimal loadings are: 0.55 g CuCl per g NaY; 0.50 g CuCl per g Cu(i)Y; 0.35 g CuCl per g 13X; 0.25 g CuCl per g 4A; 0.20 g CuCl per g γ-Al_2_O_3_. Apparently, the differences in the adsorption capacity originate from the different structure of the support. The γ-Al_2_O_3_ has the smallest surface area, which has a small dispersion capacity of CuCl, thus it has a low CO adsorption capacity (<1.09 mmol g^−1^, 293 K, 60 kPa). Also the results of Wu *et al.*^107^ fit in this trend, as they found an optimal loading of 0.33 g CuCl per g SAPO-34, corresponding to a CO adsorption capacity of 1.8 mmol g^−1^ at 298 K and 1 bar. Based on their the results, Xie *et al.*^105,106^ designed the commercialised adsorbent PU-1. This adsorbent shows a high CO adsorption capacity (>50 ml g^−1^ or 2.03 mmol g^−1^ at 1 atm and ambient temperature) and a high selectivity for CO over other gases including H_2_ (52), N_2_ (26), CH_4_ (17). The selectivity of CO over CO_2_ is modest, namely 1.93, hence CO_2_ is removed during the gas pretreatment in the PU-1 process. A large-scale plant has been designed using this adsorbent in a VPSA process and built in China for separation of CO from syngas.^54^

Nowadays, Cu(ii) salts are more often be chosen as starting material due to their chemical stability and cost. Cu(i) is usually obtained by reduction under vacuum or at elevated temperature and a suitable reducing agent. Ma *et al.*^89^ presented a method to impregnate CuCl into zeolite using a CuCl_2_–Cu(HCOO)_2_ mixture as precursors. The synthetic method is the same as was used for an AC support (Section 3.2.1). The CO adsorption ability of CuCl/Y (2.23 mmol g^−1^; the Cu loading is 4 mmol g^−1^) is as high as that of AC modified with a CuCl monolayer (2.28 mmol g^−1^). The comparison of the isosteric heat of adsorption for CuCl/Y (63.5 kJ mol^−1^) and CuCl/AC (25 kJ mol^−1^) reveals a stronger interaction between the zeolite based adsorbent and CO. As a result, CuCl/AC shows a better adsorption–desorption reversibility of CO (98% of CO desorbed in the first cycle) than CuCl/Y (70% of CO desorbed in the first cycle).

Gao *et al.*^104^ did experiments on materials similar to PU-1. Zeolite HY powder (SiO_2_/Al_2_O_3_ = 5) was mixed together with CuCl_2_·2H_2_O to prepare the CuCl/Y adsorbent. The sample is activated in CO atmosphere at 663 K to achieve full reduction of Cu(ii) to Cu(i). It is found that the amount of adsorbed CO increases greatly when the Cu(i) loading increases and the highest CO adsorption is 2.67 mmol g^−1^ (1 bar, 303 K) when the CuCl loading reaches 5.0 mmol g^−1^. The CO adsorption ability decreases when the loading of CuCl into the zeolite is further increased due to the blockage of pore channels and the corresponding decreasing accessibility of Cu(i). The CO/CO_2_, CO/CH_4_ and CO/N_2_ adsorption selectivity factors are 2.83, 24.73 and 68. A high selectivity can be obtained, due to the stronger interaction between the highly dispersed CuCl and CO *via* the π-complexation and the weaker van der Waals and electrostatic interactions of CO_2_, CH_4_ and N_2_ with the adsorbent.

Not only the available pore volume, also the nature of the countercations in the zeolite have an effect on the performance of CuCl-doped zeolites. While the above results on CuCl embedded in zeolite Y where performed on either Na^+^ or H^+^ exchanged zeolite Y, Fan *et al.*^108^ studied CO adsorption on zeolite Y, with varying amounts of Na^+^ and H^+^ countercations. They found that the CO-adsorption capacity is higher, the higher the acidity (higher amount) of H^+^ of the framework. The framework hydroxyls provided good sites for exchange of H^+^ with Cu^+^, while the pure Na^+^-Y showed the highest amount of CuCl particles around the zeolite support. Yang *et al.*^109^ compared the performance of CuCl dispersed in zeolite Y exchanged with Na^+^, La^3+^ and Ce^3+^. The CO-adsorption capacity at 1 bar and 298 K remained roughly the same (≈2.5 mmol g^−1^), but the CO/N_2_ selectivity improved from 28 (Na^+^), to 47 (Ce^3+^) and 53 (La^3+^) for the lanthanide exchanged zeolites due to a decrease in the adsorption of N_2_.

It is intriguing that the PU-1 process is a VPSA process, while several reports show that desorption by reducing the CO pressure at ambient temperature from CuCl-doped zeolites is limited. *E.g.*, the CuCl (5 mmol g^−1^)-zeolite Y studied by Gao *et al.*^104^ requires heating to 453 K and vacuum for 30 minutes to achieve complete desorption. Fan *et al.*^108^ also observed minimal CO desorption from various CuCl doped zeolite Y samples upon pressure reduction.

Very strong chemisorption was found for Cu^+^ present not in the form of CuCl, but as the countercations in ZSM-5. Rakic *et al.*^110^ exchanged HZSM-5 with Cu^2+^, Fe^2+^, Co^2+^ as well as bimetalic forms. They found for CuZSM-5 (230 mmol g^−1^ Copper) that CO-adsorption was dominated by irreversibly adsorbed CO, namely 110 μmol g^−1^ (adsorbed at a few Pa at 303 K) of a total CO-adsorption capacity around 170 μmol g^−1^. The heat of adsorption of this irreversibly bound CO has a very high value, namely 125 kJ mol^−1^. It was found that this was due to the presence of Cu^+^, due to reduction of Cu^2+^ during the pretreatment in vacuum. In fact, only in the Cu-containing materials significant irreversibly adsorbed CO was obverved. These results may indicate that CO adsorption on Cu^+^ exchanged zeolites may simply be too strong, compared to dispersed CuCl, to make this a viable adsorbent for CO separation.

In general, zeolites do show higher CO adsorption results than activated carbon due to favourable electrostatic interactions between the cation ions and CO. However, the best adsorption functionality still comes from d-metal salts, especially CuCl dispersed onto a zeolite adsorbent. The adsorption ability of metal salt impregnated zeolites is limited by its dispersion capacity and the degree of reduction of Cu(ii) to Cu(i). After reaching the loading threshold value, increasing the loading of Cu(i) further will lead to the accumulation of excessive copper and the blockage of pore channels decreasing the adsorption capacity.

#### 3.2.3 Mesoporous alumina

Mesoporous alumina adsorbents have the characteristics of large specific surface area, high dispersion of active species on the grain surface, and mesopore size with a uniform pore size distribution, which can be considered as a potential porous support for CO adsorption. In the commercialised pressure swing adsorption process developed by Kansai Coke and Chemicals Co. Ltd, the active alumina carrier has an appropriated pore size distribution, which is doped with carbon and CuCl for the chemisorption of CO. The carbon is used to prevent the oxidation of Cu(i).^53^ It is reported in two commercial CO-PSA plants, which recover CO from Linz-Donawitz converter exhaust gas (LDG: 68% CO, 16% CO_2_, 13% N_2_, 2% H_2_ and 1% O2 + Ar) from steel-making plants, that the product CO has a high purity of 99% and a high yield of 90% by applying this specific adsorbent. Further studies have continued to feature metal ion-modified alumina for CO adsorption purposes. Wang and Lin^111^ prepared CuCl-modified γ-alumina by immersing the sol–gel derived γ-alumina support into the cuprous solution under inert atmosphere. The synthesised sample absorbs CO with an adsorption capacity of 0.78 mmol g^−1^.

As described in Section 3.2.2, γ-Al_2_O_3_ has a lower surface area compared to zeolites, which result in a low dispersion of CuCl and thus a lower CO adsorption capacity. Cho *et al.*^112^ found that mesoporous boehmite (γ-AlO(OH)), decomposed aluminium hydroxide, shows a larger surface area compared to the conventional γ-alumina. The Cu(i) was introduced into the support by a heat treatment of the CuCl and boehmite mixtures under vacuum. The CO adsorption capacity of Cu(i)/boehmite (1.56 mmol g^−1^) is higher than that of their Cu(i)/γ-alumina (0.45 mmol g^−1^), which is caused by the higher hydrophilicity of boehmite. Specifically, the higher amount of hydroxyl groups on the surface of the boehmite results in a stronger affinity of CuCl, which leads to a better dispersion of CuCl in mesopores of the support. Fan *et al.* came to a similar conclusion: a higher amount of hydroxyls in zeolite Y led to better dispersion of CuCl in the pores (*vide supra*).^108^ The CuCl-boehmite showed a high CO/CO_2_ selectivity of up to 12.4 and it maintained its CO adsorption capacity even after 70 adsorption–desorption cycles.

Cho *et al.*^113^ then continued their studies using an even further decomposed aluminium oxide, bayerite, as their substrate. The material was prepared by first heat treating the bayerite under N_2_ atmosphere, after which CuCl and the treated bayerite were physically mixed at room temperature. The mixture was then heated under vacuum resulting in the adsorbent. Using a loading of 30 wt% CuCl they were able to obtain a CO adsorption capacity of up to 1.97 mmol g^−1^ and a CO/CO_2_ selectivity of 16.8. Using a higher loading of 36 wt% they were able to increase the CO/CO_2_ selectivity even further to 35.5, although the overall CO adsorption capacity decreased to 1.69 mmol g^−1^. Both of these loadings show both a higher capacity and a higher CO/CO_2_ selectivity than that of the Cu(i)/γ-alumina and Cu(i)/boehmite.

Yeom *et al.*^114^ synthesised a mesoporous alumina with a post-hydrolysis method using a chemical template. This mesoporous alumina has a maximum uptake capacity of 0.05 mmol g^−1^ at 1 bar and 25 °C. After immobilising Pd-nanodots, the uptake capacity of the adsorbent is enhanced to 0.33 mmol g^−1^ at 1 bar and 25 °C. At higher pressures, however, the uptake strongly increases to a capacity of 3.9 mmol g^−1^ at 4 bar and 25 °C. Without the Pd-nanodots the capacity at these conditions is 2.86 mmol g^−1^, only 27% lower. The CO-adsorption isotherms indeed show a type V shape, with the increase in slope starting at 2.5 bar, indicative of the adsorbate-adsorbate interactions being more favourable than the adsorbate–adsorbent interations. The size of the pores, mesopores of 3.4 nm, is probably instrumental in allowing for a high degree of favourable CO-CO interactions at high filling.

#### 3.2.4 Metal–organic frameworks (MOFs)

Metal–organic frameworks are materials that consist of metal ions or metal clusters linked together by organic ligands forming crystalline structures in 1, 2 or 3 dimensions. Due to the way the metal centers and the ligands connect, MOFs are often highly porous structures with reported BET surfaces of more than 7500 m^2^ g^−1^.^115^ The variety of MOFs available, both topologically as well as chemically, is staggering due to the large variety of metal centers and organic ligands available; over 5 million structures are reported in the MOF database published by the Snurr group.^116^

The tunable porosity and reactivity in combination with their extremely high surface area make MOFs suitable for gas separation purposes. Size exclusion separation of CO and N_2_ with MOFs is not feasible due to their similarity in kinetic diameter. What is interesting in the application of MOFs for CO separation, however, is their ability to incorporate d-metals into their structure and correspondingly capture CO *via* metal carbonyl bonds. These unsaturated metal-ions are either present in the structure of the MOF itself, such as in M-MOF-74 or Cu-BTC, or similarly to the previously discussed materials introduced as a doped π-complexation moiety, such as in CuCl in MIL-100(Fe).

An often heard argument against the usage of MOFs for industrial applications is their high cost and low stability, which would make these types of materials uneconomical and, therefore, prevent their usage in industrial-scale applications. Firstly, the stability of MOFs is highly dependent on the building blocks and their net. While going into the specifics of what constitutes a stable MOF is outside the scope of this review, several reviews have already been written on the overall design of stable MOFs and which design elements improve stability.^117–120^ While having to adhere to certain design principles does limit the total scope of possible MOFs, with millions of already known structures and more being discovered every day, this should not pose a problem that cannot be overcome. Secondly, the price of the MOFs is largely determined by the precursor materials and, especially, solvent costs associated with solvothermal methods.^121^ If aqueous or liquid assisted grinding methods are used, combined with industrial scale production of the required linkers, prices in the order of magnitude of $10 per kg of MOF could be achieved on an industrial scale.^121^

##### 3.2.4.1 Unsaturated transition metal atoms as CO adsorption sites

In literature, several CO binding mechanisms are described for different MOFs. A frequently described mechanism uses MOFs with coordinatively unsaturated metal sites, examples are MOF-74 (Co^2+^, Fe^2+^, Mg^2+^, Mn^2+^, Ni^2+^, Zn^2+^), HKUST-1 (Cu^2+^), MIL-101 (Cr^3+^, Fe^3+^), MIL-100 (Cr^3+^), and DUT-82 (Rh^2+^).

**Fig. 8:**
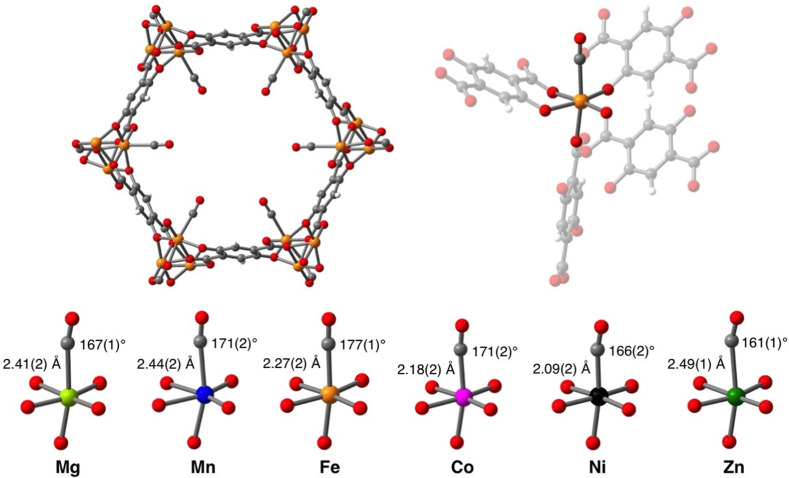
Structures of M-MOF-74 determined by neutron diffraction. Top left: View along the *c*-axis of Fe-MOF-74·1.5 CO, corresponding with 75% loading. Top right: Coordination environment of Fe-MOF-74·1.5 CO Bottom: First coordination sphere of the M^2+^-ions in M-MOF-74·1.5 CO. M–CO distance and M–C–O angles are indicated. Reprinted with permission from ref. 122. Copyright 2014 American Chemical Society.

Likely the most studied MOF with unsaturated metal ions, MOF-74 (also termed CPO-27-M or M_2_(dobdc), M referring here to the specific metal ion used) consists of 2,5-dioxido-1,4-benzenedicarboxylate (dobdc) linkers coordinated to divalent metal cations (Mg^2+^, Mn^2+^, Fe^2+^, Co^2+^, Ni^2+^ and Zn^2+^) (Fig. 8). The metal center is coordinated to five oxygen atoms from four dobdc and one H_2_O in an octahedral fashion. The extended MOF structure has a network of one-dimensional honeycomb-shaped channels with around 11 Å diameter. By removing the coordinated water molecules with heat or reduced vapour pressure, the unsaturated metal sites can bind to CO *via* σ- and π-type bonding *via* the mechanism described in Section 2.2.2. CO adsorption follows the Irving-Williams series with the highest (negative) isosteric heats of adsorption following Ni > Co > Fe > Mg > Mn > Zn, ranging from −52.7 to −27.2 kJ mol^−1^. In line with these values, the M–CO bond length increases from Fe to Zn. The strong Ni(ii)–CO bond, and accompagnying short Ni–C bond length of 2.148 Å is due to the large charge transfer and the π-back donation effect of Ni(ii). Unlike Ni(ii) metal sites, the Mg(ii) ions do not possess d-electrons for π-back-bonding, and Zn(ii) ions with a filled d-shell cannot receive σ-donation with fully occupied 3d-orbitals. Therefore, the M–CO interactions in M-MOF-74 (M = Mg and Zn) are weak, and the Mg–C and Zn–C distances (2.486 and 2.491 Å) are long. The adsorption capacities at 1.2 bar for the three best performing materials are in fact close to 1 CO per M(ii), which would be, roughly, 6 mmol g^−1^, namely 5.78 mmol g^−1^ for Ni(ii), 5.90 mmol g^−1^ for Co(ii) and 5.95 mmol g^−1^ for Fe(ii). The reported IAST selectivity for Ni-MOF-74 of 2000–5000 (CO/H_2_) and 155–500 (CO/N_2_) are the highest reported values in literature, while the capacity of 5.79 mmol g^−1^ at 1.2 bar CO at 298 K is one of the highest reported values for MOFs under these mild conditions.^122^ Yet, the dynamic CO-adsorption capacity (defined as difference in amount of adsorbed CO at 1 *versus* 0.1 bar at 298 K), the strongly adsorbing M(ii)-MOF-74 are actually outperformed by Mg(ii)-MOF-74, which has a dynamic CO adsorption capacity of 3.13 mmol g^−1^.

The dynamics of carbon monoxide adsorbed in M-MOF-74 (M = Mg and Zn) is investigated by Lucier *et al.*^123^ using solid state NMR spectroscopy together with a spectral simulation approach. The motion of carbon monoxide adsorbed in open metal sites of MOF-74 are dominated by two different modes: a localised “wobbling” motion of CO at a specific metallic center and a non-localised sixfold (C_6_) “hopping” motion of CO transferring between adjacent open metallic centers. It is demonstrated that a larger wobbling and smaller hopping angles lead to weaker CO–metal binding by comparing the heats of adsorption and CO motional angles.

**Fig. 9:**
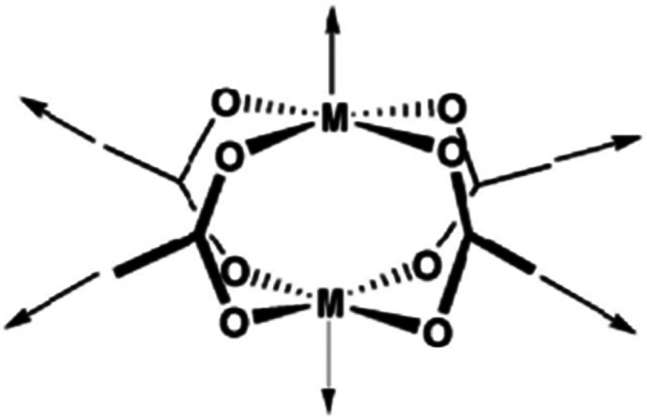
General structure of an M^2+^–M^2+^ paddlewheel. Reprinted with permission from ref. 125. Copyright 2008 American Chemical Society.

As the most studied MOF with unsaturated metal sites, activated HKUST-1 (Cu_3_(BTC)_2_; BTC = 1,3,5-benzenetricarboxylate) was also reported by Yin *et al.*^124^ to have a CO adsorption capacity of 0.30 mmol g^−1^ and an IAST selectivity in equimolar streams of 8.3 (CO/H_2_) and 1.5 (CO/N_2_) at 25 °C, 1 bar. HKUST-1 possesses Cu^2+^–Cu^2+^ paddlewheel units, the general structure of which is shown in Fig. 9.

Similar to HKUST-1, the unsaturated metal sites in the Rh^2+^-Rh^2+^ paddlewheel units of activated Rh-DUT-82 can also bind to CO molecules. It is reported that this framework has a CO uptake of 2.11 mmol g^−1^ at 25 °C and 1 bar, which is higher than the CO adsorption capacities of HKUST-1 under the same conditions^126^ The adsorption enthalpy is also quite high (−50.6 kJ mol^−1^). The CO adsorption has not been compared with other gasses.

**Fig. 10:**
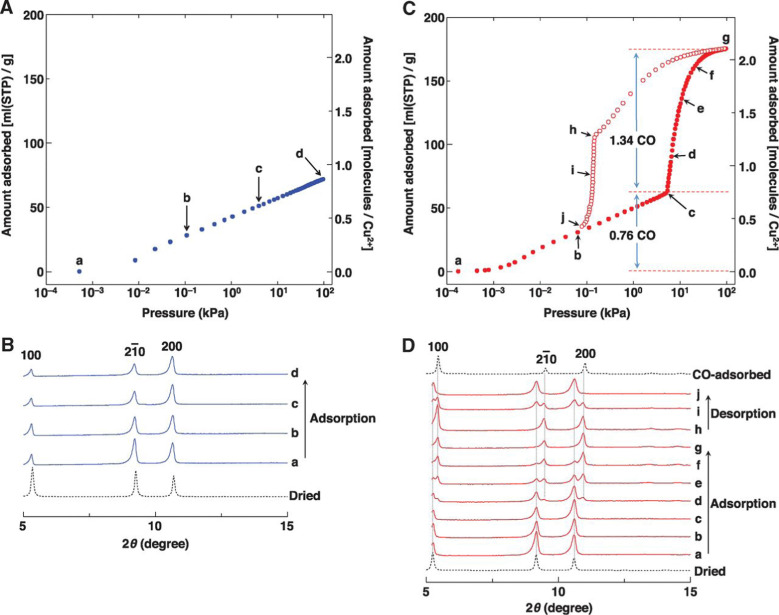
N_2_ and CO adsorption isotherms and coinciding PXRD measurements. (A) N_2_ adsorption isotherm at 120 K. (B) PXRD patterns of the measurement points indicated in A (a–d), with the simulated pattern of dried Cu-aip on the bottom. (C) CO adsorption (•) and desorption (°) isotherms at 120 K. (D) PXRD patterns of the measurement points indicated in A (a–j), with the simulated pattern of dried Cu-aip on the bottom and CO adsorbed Cu-aip at the top. From ref. 127. Reprinted with permission from AAAS.

A study by Sato *et al.*^127^ demonstrated the self-accelerating CO adsorption (7.15 mmol g^−1^ at 120 K, 1 bar) in a Cu(ii) porous coordination polymer (PCP) (Cu(aip)) as a result of the conformational change of the structure upon adsorption of CO (Fig. 10). The MOF contains paddlewheel units constructed by Cu^2+^ and 5-azidoisophthalate (AIP). After activation, the bound water molecules were removed from the axial position of the Cu–Cu paddlewheel unit, which narrows the channels of the framework and reduces the accessible pore volume from 38% of the unit cell to 25%. The structure can be changed into a third phase, similar to the as-synthesised crystal structure, by exposing the dry Cu(aip) to enough CO. This phase can be seen in Fig. 10C and D between points c and h. Water can similarly be used to return to the as-synthesised structure, effectively undoing the drying. The dry Cu-PCP shows a selectivity of 2.24 towards CO in CO/N_2_ mixtures at 120 K. When CO diffuses in the small channel of the Cu-PCP, it breaks a coordination bond between Cu and the O atoms of carboxylates and forms a coordination bond with Cu^2+^. N_2_, on the other hand, due to its extremely weak coordination ability, cannot diffuse into the channel which is filled by the coordinated CO. The MOF demonstrated a decent capacity for concentrating CO to 84% in one cycle from 1 : 1 CO/N_2_. However, the tests were performed at 120 K, complicating comparison with other materials. However, the low CO/N_2_ selectivity of 2.24 is indicative of relatively weak interactions with CO.

The adsorption of CO on MIL-100(Fe) was studied by Yoon *et al.*^128^ The oxidation states of iron and the amount of open metal sites were quantified using *in situ* IR spectroscopic analysis under CO atmosphere. At room temperature, CO molecules interact weakly with the Fe^3+^ sites in the network as confirmed by a weak band at 2190 cm^−1^ and an adsorption enthalpy of −39 to −28 kJ mol^−1^. By increasing the activation temperature, two new bands at 2182 and 2173 cm^−1^ appeared, which are assigned to the adsorption on the open Fe^2+^ sites. It is demonstrated that uncoordinated Fe^2+^ sites are created at higher temperature due to the loss of an X^−^ ligand and partial reduction of Fe^3+^–X^−^ sites (X^−^ = F^−^ or OH^−^). The reduced sample with both open Fe^2+^ and Fe^3+^ sites shows a higher adsorption enthalpy of −51 to −39 kJ mol^−1^, which validate that the Fe^2+^ sites have a stronger interaction with the CO molecules.

Preferential binding often occurs already at low CO partial pressures (<0.1 bar) evidenced by steep adsorption isotherms. As more metal sites are occupied, the adsorptio n slows down due to increased scarcity of sites. After all metal sites are occupied the adsorption is limited to physisorption. At high pressures CO adsorption does still occur, but dispersion forces dominate causing a severe drop in CO selectivity.

##### 3.2.4.2 Spin-crossover

Other CO adsorption mechanisms include binding by spin state transition of the metal in the framework FeBTTri; (Fe_3_[(Fe_4_Cl)_3_(BTTri)_8_]_2_·18CH_3_OH with H_3_BTTri = (1,3,5-tris(1*H*-1,2,3-triazol-5-yl)benzene)) upon coordination of CO studied by Reed *et al.*^129^ After the removal of methanol, the coordinatively unsaturated Fe^2+^ in this MOF binds carbon monoxide through a unique spin state change mechanism: the square pyramidal, high-spin Fe^2+^ centres switch to octahedral, low-spin Fe^2+^ centres upon CO coordination. The Fe^2+^ sites revert back to the high-spin ground state upon the desorption of carbon monoxide, enabling the facile reversibility of the material. The sample shows a high CO uptake at 298 K of 2.25 mmol g^−1^ at 0.15 mbar and 2.7 mmol g^−1^ at 0.27 bar due to the strong interactions between the low-spin state Fe^2+^ and CO. Highly selective binding towards CO over other gas molecules is shown by high IAST selectivities towards CO at 298 K and 1 bar in a 1 : 1 mixture with gases such as H_2_ (1500), N_2_ (250), CO_2_ (28), CH_4_ (110), and other hydrocarbons (C_2_H_6_ (16) and C_2_H_4_ (27)), while retaining good reversibility. It is reported that the initial slope of the adsorption isotherm (below 0.22 bar) is steep due to strong metal–CO interactions (Δ*H*_CO_ = −65 kJ mol^−1^) and 75% of the preferential binding sites are occupied. Above this pressure, weak interactions were responsible for CO adsorption. The authors also claim that the sample is suitable for scavenging trace amounts of CO.

**Fig. 11:**
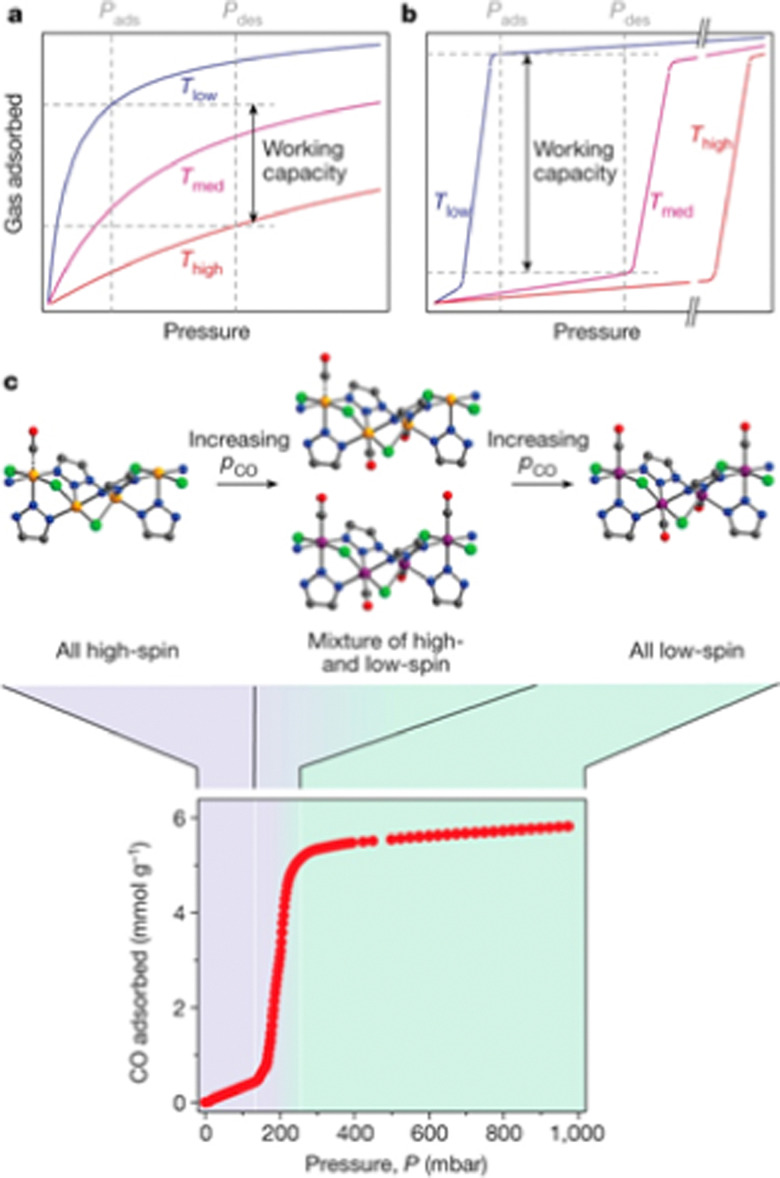
(a and b) Schematic of how working capacity is increased due to the sigmoidal adsorption curve caused by the spin crossover; (c) schematic view of the mechanism of the spin crossover in Fe_2_Cl_2_(bbta) due to the adsorption of CO. Colour code of the atoms in the structures shown: Fe (yellow), Cl (green), N (blue), C (grey), O (red). Reprinted by permission from Springer Nature,^130^ copyright 2017.

Another material that utilises such a spin-crossover to achieve a cooperative binding effect for CO is the metal–organic framework Fe_2_Cl_2_(bbta) as presented by Reed *et al.*^130^ It consist of helical Fe^2+^, Cl^−^ chains linked with each other *via* stiff, aromatic benzo(1,2-*d*:4,5-*d*)bistriazole^2−^ (bbta) linkers that bond coordinatively to the Fe^2+^ ions, resulting in Fe^2+^ ions with one unsaturated octahedral site, meaning the coordination is square pyramidal. This results in a honeycomb pattern of hexagonal tubes with the unsaturated sites being available at the inside of the corner points of the hexagonal pore cross-section. The adsorption isotherm of CO on Fe_2_Cl_2_(bbta) has a sigmoidal-like shape which allows for an improved working capacity compared to regular Langmuir-like adsorption isotherms (Fig. 11). This sigmoidal-like isotherm is caused by the change from a Fe^2+^ high spin state to a low spin state which is caused by the adsorption of CO. The spin change is shown to be dependent on two factors: temperature and the CO partial pressure. The adsorption of CO onto the metal site is the factor that changes the spin state, while the temperature affects the location of the adsorption step and the isotherm shape. Together these factors allow for a thermally switchable CO adsorbent which utilises the spin-crossover effect to obtain a more favourable isotherm shape. Fe_2_Cl_2_(bbta) is an attractive CO adsorbent as it has both a high capacity of CO chemisorption of 5.87 mmol g^−1^, as well as a high selectivity of 85 and 232 towards CO compared to N_2_ and H_2_, respectively, at a CO : H_2_/N_2_ ratio of 1 : 3 at 1 bar of total pressure.

##### 3.2.4.3 Metal salts supported on metal–organic frameworks

Inspired by the performance of adsorbents like alumina, activated carbon and zeolites impregnated with Cu(i) salts, this route has also been explored with metal–organic frameworks as substrates. The size and geometry of pores and the surface area can be tuned much more easily for MOFs compared to the other aforementioned porous materials, making it a promising candidate as porous carrier.

Yin *et al.*^124^ used the dry grinding method to impregnate HKUST-1 with CuCl. The impregnation of 10 wt% CuCl resulted in an increased adsorption capacity of 0.54 mmol g^−1^ compared to the bare HKUST-1 capacity of 0.30 mmol g^−1^, as well as an increased IAST selectivity in equimolar streams of 66.4 (CO/H_2_) and 9.5 (CO/N_2_) at 25 °C, 1 bar compared to the selectivity values of the bare HKUST-1 of 8.3 (CO/H_2_) and 1.5 (CO/N_2_).

Peng *et al.*^131^ developed a wet impregnation method to uniformly insert the Cu(ii) salts on the MIL-100(Fe) network using CuCl_2_ and Cu(HCOO)_2_ as starting materials, the Cu(ii) is then reduced to Cu(i) under vacuum at 423 K. The CO adsorption capacity increases (from 0.38 to 2.78 mmol g^−1^ at 298 K and 1 bar) by increasing the CuCl loading (from 0 to 8 mmol CuCl per g adsorbent). Also the CO/N_2_ adsorption selectivity of MIL-100(Fe) loaded with 8 mmol g^−1^ is with 169 also much higher than that of the original network (1.5). In accordance with the high selectivity, CuCl-incorporated MIL-100(Fe) has a high isosteric heat of adsorption of −50 kJ mol^−1^ for CO. The regeneration and stability of the absorbent are not mentioned.

Li *et al.*^132^ used a double solvent method to impregnate and reduce Cu(i) in MIL-101(Cr) to prevent the aggregation of Cu(i) at the outside surface of the MOF. They placed the MIL-101(Cr) in hexane and added an aqueous CuCl_2_ solution with a volume roughly equal to the pore volume of MIL-101(Cr) dropwise to the MOF suspension under stirring. After leaving the suspension to stir for 3 hours, the particles were allowed to settle, the solvent was decanted and the particles were left to dry under vacuum at 373 K for 12 hours. For the reduction, the MIL-101(Cr) was again placed in hexane and an amount of aqueous Na_2_SO_3_ solution equimolar to the amount of Cu(ii) was added dropwise under stirring. The sample was then filtrated, washed with water and dried under vacuum at 373 K for 12 hours. Plain MIL-101(Cr) showed a CO adsorption capacity of 1.33 mmol g^−1^, while the highest adsorption capacity of 2.42 mmol g^−1^ was reached with a loading of 3.14 mmol g^−1^ Cu(i)@MIL-101(Cr), both at 298 K and 1 bar. Compared to the traditional wet-impregnation and liquid reduction, the samples prepared *via* the double solvent method showed higher surface area, higher pore volume and weaker CuCl XRD peaks, all indicating a better dispersion of CuCl compared to the conventional methods of wet impregnation and liquid reduction.

For practical application, the Cu(i) incorporated MOFs should not only exhibit high CO adsorption capability, but also good air stability, which may be compromised by Cu(i) being easily oxidised. Inspired by the commercialised COSORB process, Wang *et al.*^133^ introduced CuAlCl_4_ complex into the MIL-101(Cr) framework. The adsorbent exhibits the highest CO uptake capacity of (2.17 mmol g^−1^) at 298 K and a CO/N_2_ selectivity of 32 when loaded with 5 mmol CuAdlCl_4_/g MIL-101. The adsorbent can be regenerated under 4 mmHg at room temperature, after which the original adsorption capacity is recovered. It also exhibits good stability: most of its CO adsorption capacity can be retained after expose to air for a month. This is due to the molecular structure of CuAlCl_4_ and to the interactions between Cl and Cu that prevent the reduction of Cu(i).

Another way to increase the stability of the adsorbents is using promoters such as vanadium or zinc species. A CuV-loaded MIL-101(Cr) is synthesised using both Cu(NO_3_)_2_ and VCl_3_ as precursors. The vanadium ions are used to facilitate the reduction of the Cu(ii). Because the synergetic effect between Cu and V, the Cu(ii) can be reduced into Cu(i) at a relatively low temperature of 523 K. Note that MOFs tend to collapse at high temperature and lose their porosity. Moreover, the adsorbent shows good CO selectivity and stability, retaining up to 91.8% of the original capacity for 2 weeks upon exposure to atmospheric air due to the assistance of the vanadium species and its preferential oxidation over the Cu(i) sites in the sample.^134^ The Cu(i)Zn co-doped MIL-100(Fe) adsorbent has a CO working capacity of 1.45 mmol g^−1^ and a high CO/CO_2_ selectivity of 104.^135^

Some MOFs that show relatively poor performance in their pure form, do gain improved CO adsorption capacities when impregnated with Cu(i). However, impregnation of Ni-MOF-74, which shows the highest CO adsorption capacity due to the unsaturated metal sites, with various Cu-salts did not result in dramatic differences with an overall increase of 0.5 mol g^−1^ in dynamic capacity and a 10% increase in mol_CO_/mol_metal_ compared to the pure Ni-MOF-74. This result is in stark contrast to the results obtained with ACs and zeolites and some other MOFs (*e.g.*, (Fe-MIL-100)), where the increase in capacity can double or more. This effect is caused by the Cu-moieties competing with Ni-sites for space accessible for CO molecules, reducing the effectiveness of the added Cu-sites.

In conclusion, the use of Cu(i) salt could significantly increase the CO adsorption uptake of the metal–organic frameworks. However, the Cu^+^ ion is sensitive to air and water, so in order to avoid the oxidation and hydrolysis, all operations, including preparation and storage, must be performed under dry and inert atmosphere. Though the use of complexes like CuAlCl_4_, CuNO_3_–VCl_3_ or CuClZnCl_2_ has been shown to significantly slow down the Cu(i) oxidation.

#### 3.2.5 Evaluative notes

The aforementioned research show that it is difficult to directly use the conventional ACs, zeolites or mesoporous alumina as CO adsorbents, because of their low adsorption capacity and low selectivity for CO (<0.5 mmol g^−1^ at 298 K, 1 bar). MOFs with unsaturated metal sites, on the other hand, *e.g.*, (MOF-74) display the best intrinsic CO adsorption performance with a maximum capacity of 6.06 mol g^−1^ and selectivities of 2000–5000 (CO/H_2_) and 155–500 (CO/N_2_) belonging to Ni-MOF-74.

Even though MOFs with unsaturated metal sites show excellent CO adsorption capacities and selectivities, there are also some drawbacks when they are used in real process conditions. Open metal sites can also strongly interact with water, causing the MOF (*e.g.*, HKUST-1) to degrade structurally by hydrolysis. Water adsorption to these sites also results in a competitive adsorption mechanism, which would severely impact the adsorbent capacity. Therefore, competitive adsorption of CO and H_2_O needs to be studied in these materials. Besides the impact of water, long-term exposure to oxygen also causes MOFs, where the transition metal is not in its highest oxidation state (like MOF-74 with M = Fe(ii), Co(ii) or Mn(ii)), to lose their high adsorption capacity due to oxidation of these metal centres.

Another widely followed strategies is to use ACs, zeolites, mesoporous alumina or MOFs as porous supports to achieve a high dispersion of metal salts, especially CuCl. The advantage is that the π-complexation between Cu(i) ions and CO, which is much stronger than the van der Waals forces or electrostatic interaction between the adsorbents and other gases, can help to achieve high adsorption capacity and high selectivity of CO. The Kobe Steel and PU-1 PSA processes utilise CuCl as the active material, which is sensitive to O_2_ and moisture. For example, after exposing zeolite Y in which 5 mmol g^−1^ CuCl is dispersed for 24 hours to ambient air, the CO adsorption capacity decreased by almost 90 percent.^104^ The original CO adsorption capacity could be regained exposing the material to a reducing CO atmosphere at 663 K. To circumvent the precarious handling of CuCl, recent studies are focused on employing Cu(ii) salts, which are stable in air, as precursors. However, it is still a challenge to control the reduction of Cu(ii) into Cu(i) in a precise way. Meanwhile, it is of importance to choose suitable porous supports, which should have a good stability, a high surface area, and a suitable pore size to allow for high CuCl loading, as the more CuCl sites are dispersed, the higher the CO adsorption capacity will be. It is shown from the previous discussion on activated carbon^90,91^ and mesoporous alumina^112,113^ (Section 3.2.1 and 3.2.3) that a more hydrophilic surface will allow for a higher loading of CuCl with good dispersion. A similar concept based on hydrophilicity to increase the dispersion of Cu(i) was also utilised in MIL-101(Cr) *via* the impregnation and reduction of Cu(ii) with a double solvent method.^132^ Attempts have also been made to stabilise the Cu(i) by co-impregnating a sacrificial metal such has been done in MOFs with CuAlCl_4_,^133^ CuNO_3_–VCl_3_,^134^ or CuClZnCl_2_.^135^ A recent review on CO adsorption by Feyzbar-Khalkhali-Nejad *et al.*^12^ went into various other examples of non-standard methods of transition metal impregnation, stabilisation, and reduction in porous supports. To conclude, the stability of Cu(i), which is now mainly used as active metal site, remains a challenge for the reliable preparation of high surface area adsorbents, and means that the adsorbents can only be used for dry, probably oxygen free gas streams. The introduction of other π-complex active metal ions into porous supports can lead to other promising adsorbents, but the variety of active metal ions that has been investigated is very limited.

All in all, adsorbents have been identified with significantly higher CO-adsorption capacities (up to 6 mmol g^−1^ at 298 K, 1 bar) compared to only 1–2 mmol g^−1^ at the same conditions for the commercially used adsorbents (see Section 2.4.4). The reported CO/N_2_ selectivities regularly are several 100's compared to 20–25 for the commercial adsorbents. Generally, many of the adsorbents that are promising in terms of CO/N_2_ selectivity show strong physisorption up to chemisorption of CO (from −20 to −80 kJ mol^−1^). As discussed above, N_2_ and CO possess similar properties, rendering it difficult to achieve decent selectivity of CO and N_2_ based on weak physisorption. Indeed, Table 3 shows that high CO/N_2_ selectivity is associated with a higher adsorption enthalpy, which needs to be energetically overcome during the desorption stage.

**Table 3:** A comparative overview of the adsorbents for CO separation process

Material	SA^BET^ [m^2^ g^−1^]	Equilibrium CO capacity [mmol g^−1^]	*T*/*P* [K bar^−1^]	Dynamic CO capacity^b^ [mmol g^−1^]	Enthalpy of adsorption [−kJ mol^−1^]	CO/N_2_ selectivity^c^	CO/H_2_ selectivity^c^	CO/CO_2_ selectivity^c^	Stability	Regeneration	Ref.
Activated carbons											
AC	1170	0.2	298/1	—	—	—	—	—	—	—	89
CuCl (4 mmol g^−1^)-AC	652	2.28	298/1	1.09	25	35^c^	—	3.2^c^	Sensitive to air	—	89
AC	1784	0.25	303/1	—	—	—	—	—	—	—	87
CuCl (7 mmol g^−1^)-AC	478	2.95	303/1	—	—	44.6^c^	—	6.2^c^	Sensitive to air and moisture	No loss of capacity after 6 cycles	87
AC	1082	0.28	298/1	0.23	30–15	—	—	—	—	—	92
CuCl (4 mmol g^−1^)-AC	505	1.85	298/1	0.94	50–47	34.3^d^	—	2.6^d^	—	—	92
AC	—	0.35	313/1	0.31	—	—	0.19^c^	12^c^	—	—	90
CuCl (0.5 mmol g^−1^)-AC	—	0.54	313/1	0.38	—	—	0.31^c^	20^c^	—	—	90
CuCl (2 mmol g^−1^)-AC	—	1.25	313/1	0.73	—	—	1.15^c^	73.5^c^	—	—	90
CuCl (3.5 mmol g^−1^)-AC	—	1.69	313/1	1.05	—	—	2.25^c^	121^c^	—	—	90
CuCl (5 mmol g^−1^)-AC	—	2.01	313/1	1.30	—	—	4.47^c^	201^c^	—	—	90
Ni(0)-PACS	1164	6.56	308/1	0.33	—	—	—	—	—	95% of max capacity after 10 cycles	93
Other porous carbon-based materials											
SNW-1	719	0.35	293/1	0.31	—	—	—	0.10^d^	—	—	95
0.3Cu(i)@SNW-1	533	0.77	293/1	0.45	—	—	—	0.28^d^	—	—	95
0.5Cu(i)@SNW-1	373	1.07	293/1	0.56	—	—	—	1.28^d^	—	—	95
0.7Cu(i)@SNW-1	238	1.07	293/1	0.56	—	—	—	3.84^d^	—	—	95
0.9Cu(i)@SNW-1	196	1.06	293/1	0.59	—	—	—	8.06^d^	—	—	95
1.1Cu(i)@SNW-1	168	1.04	293/1	0.60	—	—	—	11.7^d^	—	—	95
1.3Cu(i)@SNW-1	136	0.98	293/1	0.61	—	—	—	22.5^d^	—	—	95
1.5Cu(i)@SNW-1	116	0.93	293/1	0.60	—	—	—	23.3^d^	—	—	95
Zeolites											
5A	—	1.21	298/1	0.85	69-15	—	—	—	—	—	100
13X	—	0.47	298/1	0.37	70-36	—	—	—	—	—	100
ZSM-5 (SiO_2_/Al_2_O_3_ = 25)	371	0.93	303/1	0.66	33	1.8^c^	—	—	—	—	98
ZSM-5 (SiO_2_/Al_2_O_3_ = 40)	379	0.66	303/1	0.49	30	1.7^c^	—	—	—	—	98
ZSM-5 (SiO_2_/Al_2_O_3_ = 100)	348	0.45	303/1	0.35/UC	28	1.6^c^	—	—	—	—	98
ZSM-5 (SiO_2_/Al_2_O_3_ = 400)	334	0.28	303/1	0.24	25	1.3^c^	—	—	—	—	98
ZSM-5 (SiO_2_/Al_2_O_3_ = 900)	315	0.27	303/1	0.24	23	1.3^c^	—	—	—	—	98
NaX	721	1.14	303/1	0.99	26.1	2.78^c^	—	—	—	—	102
LiX	759	1.40	303/1	1.20	31.2	2.41^c^	—	—	—	—	102
KX	537	0.60	303/1	0.53	21.3	2.23^c^	—	—	—	—	102
RbX	550	0.43	303/1	0.38	20.1	1.95^c^	—	—	—	—	102
CsX	423	0.37	303/1	0.32	19.2	2.08^c^	—	—	—	—	102
NaX	692	1.14	303/1	0.97	26	2.78^c^	—	—	—	—	103
Mg(70)NaX	711	0.78	303/1	0.28	17	2.00^c^	—	—	—	—	103
Ca(95)NaX	704	1.88	303/1	0.59	33	1.80^c^	—	—	—	—	103
Sr(95)NaX	622	1.87	303/1	1.07	31	2.37^c^	—	—	—	—	103
Ba(85)NaX	565	1.23	303/1	0.94	30	2.39^c^	—	—	—	—	103
13X	—	0.39	303/0.5	—	—	—	—	0.09^c^	—	—	106
CuCl (0.523 g g^−1^)-13X	—	3.41	303/0.5	—	—	—	—	1.86^c^	—	—	106
H^+^Y (Si/Al = 2.4)	—	0.53	303/0.5	—	—	—	—	0.11^c^	—	—	106
CuCl (0.554 g g^−1^)-H^+^Y (Si/Al = 2.4)	—	3.22	303/0.5	—	—	—	—	1.92^c^	—	—	106
Na^+^Y (Si/Al = 5)	694	0.21	303/1	—	—	—	—	—	—	—	104
CuCl (5.0 mmol g^−1^)-Na^+^Y (Si/Al = 5)	329	2.72	303/1	1.15	28	65^c^	—	2.8^c^	Sensitive to air	—	104
PU-1	—	2.36	293/0.75	—	—	26^c^	52^c^	1.93^c^	—	—	105
4A	—	0.40	293/0.6	—	—	—	—	—	—	—	105
CuCl (0.25 g g^−1^)-4A	—	1.96	293/0.6	—	—	—	—	—	—	—	105
13X	—	0.58	293/0.6	—	—	—	—	—	—	—	105
CuCl (0.35 g g^−1^)-13X	—	2.94	293/0.6	—	—	—	—	—	—	—	105
Na^+^Y	—	0.48	293/0.6	—	—	—	—	—	—	—	105
CuCl (0.55 g g^−1^)-Na^+^Y	—	3.66	293/0.6	—	—	—	—	—	—	—	105
Cu^+^Y	—	1.45	293/0.6	—	—	—	—	—	—	—	105
CuCl (0.50 g g^−1^)-Cu^+^Y	—	4.05	293/0.6	—	—	—	—	—	—	—	105
SAPO-34	573	0.34	298/1	0.30	—	1.63^c^	—	—	—	—	107
CuCl (0.1 g g^−1^)-SAPO-34	481	0.39	298/1	0.25	—	2.32^c^	—	—	—	—	107
CuCl (0.3 g g^−1^)-SAPO-34	361	1.04	298/1	0.27	—	11.3^c^	—	—	—	—	107
CuCl (0.4 g g^−1^)-SAPO-34	323	1.30	298/1	0.35	—	16.0^c^	—	—	—	—	107
CuCl (0.6 g g^−1^)-SAPO-34	161	1.84	298/1	0.53	—	19.3^c^	—	—	—	—	107
NaY	901	0.63	298/1	—	—	2.6^c^	—	—	—	—	109
CuCl (27.7 wt%)-NaY	222	2.62	298/1	—	—	29.1^c^	—	—	—	—	109
CeY	761	0.30	298/1	—	—	2.3^c^	—	—	—	—	109
CuCl (28.0 wt%)-CeY	223	2.54	298/1	—	—	50.8^c^	—	—	—	—	109
LaY	768	0.30	298/1	—	—	2.3^c^	—	—	—	—	109
CuCl (29.5 wt%)-LaY	214	2.64	298/1	—	—	52.8^c^	—	—	—	—	109
Mesoporous alumina											
MA	334.2	0.04	298/1	—	—	—	—	—	—	—	114
Pd/MA	—	0.34	298/1	—	—	—	—	—	—	—	114
γ-Al_2_O_3_	—	0.05	293/0.6	—	—	—	—	—	—	—	105
CuCl (0.20 g g^−1^)-γ-Al_2_O_3_	—	1.09	293/0.6	—	—	—	—	—	—	—	105
CuCl/Boehmite	203	1.56	293/1	0.56	—	—	12.4^c^	—	—	—	112
CuCl (30 wt%)/Bayerite	302	1.97	293/1	1.17	—	49.3^c^	49.3^c^	15^c^	—	—	113
CuCl (36 wt%)/Bayerite	—	1.69	293/0.95	—	—	—	—	31.7^c^	—	—	113
Metal–organic frameworks											
MOF-5	—	0.17	298/1	0.14	16	—	—	—	—	—	100
MOF-177	—	0.12	298/1	0.08	22-18	—	—	—	—	—	100
Cu(ii)-HKUST-1	1070	0.30	298/1	0.25	—	8.3^d^	1.5^d^	—	—	—	124
CuCl (10 wt%)/Cu(ii)-HKUST-1	855	0.54	298/1	0.29	62-50	66.4^d^	9.5^d^	—	—	—	124
Cu(ii)-MOF-74	1455	0.91	300/1	0.4	—	6^d^	—	—	Sensitive to air and moisture	—	136
Cu(ii)-TDPAT	2633	1.23	300/1	1.0	—	5^d^	—	—	Sensitive to air and moisture	—	136
Cu(i)-MFU-4L	347	0.41	300/1	0.28	—	96^d^	—	—	Sensitive to air and moisture	—	136
Ni(ii)-MOF-74	1574^a^	5.78	298/1	0.84	52.7	216^d^	1705^d^	—	—	—	122
Co(ii)-MOF-74	1433^a^	5.90	298/1	1.34	48.8	163^d^	1040^d^	—	—	—	122
Mg(ii)-MOF-74	1957^a^	4.28	298/1	3.13	35.4	10^d^	170^d^	—	—	—	122
Mn(ii)-MOF-74	1797^a^	2.96	298/1	2.43	29.7	10^d^	86^d^	—	—	—	122
Fe(ii)-MOF-74	1535^a^	5.95	298/1	2.61	43.6	68^d^	507^d^	—	—	—	122
Zn(ii)-MOF-74	1105^a^	1.72	298/1	1.47	27.2	7.5^d^	47^d^	—	—	—	122
Fe(ii)Cl_2_-BBTA	1055	5.83	298/1	5.40	65.4-38.0	47^d^	128^d^	—	—	—	130
Fe(ii)Cl_2_-BTDD	1897	3.38	298/1	3.26	61.3-34.5	—	—	—	—	—	130
Fe(ii)-BTTri	1630	—	298/1	—	75.0-65.0	250^d^	1500^d^	28^d^	—	No loss of capacity after 10 cycles	129
Cu(ii)(aip)	—	7.15	120/1	1.79	19.0	2.24^c^	—	—	Sensitive to moisture	—	127
Rh(ii)-DUT-82	780	2.79	298/1	0.73	50-6	—	—	—	Thermally stable up to 250 °C	2nd and 3rd cycle show lower, constant capacity compared to the 1st cycle	126
InOF-1	—	—	—	—	52.7-36.4	—	—	—	—	—	137
Fe(ii)-MIL100	2042	0.38	298/1	0.33	39	1.5^d^	4.44^d^	0.16^d^	—	—	131
CuCl (0.8 g g^−1^)-Fe(ii)-MIL100	762	2.78	298/1	1.26	49.5	17.5^d^	55.8^d^	3.9^d^	—	—	131
Fe(ii)-MIL-100	2458	1.2	303/1	—	—	—	—	—	—	—	138
CuCl (0.9 g g^−1^)-@Fe(ii)-MIL-100	898	3.52	303/1	1.61	—	677^d^	—	29^d^	—	CO adsorption capacity is maintained during 30 cycles of CO adsorption and desorption	138
CuCl (40 wt%)-Cr(iii)-MIL-101	1030	2.82	298/1	—	—	315^d^	—	34^d^	Thermally stable up to 600 °C	Stable during 6 cycles	139
Cr(iii)-MIL-101	3788	0.50	298/1	0.44	44.5-11.7	6.3^d^	16.0^d^	—	—	—	134
CuNO_3_–VCl_3_ (2.5 mmol g^−1^) Cr(iii)-MIL-101	1697	1.19	298/1	0.62	52.0-20.0	70.1^d^	641.7^d^	—	91.8% efficient after 2 weeks of air exposure	Stable during 5 cycles	134
CuAlCl_4_ (5.0 mmol g^−1^) Cr(iii)-MIL-101	2391	2.18	298/1	1.13	—	31.5^d^	—	—	87.5% capacity after 4 weeks of exposure	Stable during 5 cycles	133
Cr(iii)-MIL-101	3615	1.33	298/1	0.74	—	4.8^c^	—	—	—	—	132
CuCl (0.83 mmol g^−1^) Cr(iii)-MIL-101	2045	1.55	298/1	0.90	—	6.7^c^	—	—	Sensitive to air	—	132
CuCl (1.75 mmol g^−1^) Cr(iii)-MIL-101	1908	1.75	298/1	1.03	—	9.2^c^	—	—	Sensitive to air	—	132
CuCl (3.14 mmol g^−1^) Cr(iii)-MIL-101	1843	2.42	298/1	1.19	—	18.6^c^	—	—	Sensitive to air	—	132
CuCl (4.38 mmol g^−1^) Cr(iii)-MIL-101	1070	2.15	298/1	0.8	—	26.9^c^	—	—	Sensitive to air	—	132
Cu (4 Cu/Ni)-Ni-MOF-74	1047	—	298/1	—	—	27^c^	—	3^c^	—	—	140
Cu (2 Cu/Co)-Co-MOF-74	1204	—	298/1	—	—	—	—	—	—	—	140
CuClZnCl_2_ (10 wt%)-MIL-100(Fe)	342.9	3.16	298/1	1.45	58	—	—	104^d^	Resistant to air	Stable during 5 cycles	135

a SA^Langmuir^.

b Defined as the difference in capacity between 1 and 0.1 bar at the given temperature.

c Idealised selectivity of an equimolar mixture given by comparison of the capacities of the two adsorbates at the given temperature and pressure.

d IAST selectivity of an equimolar mixture at the given temperature and pressure.

While there are a series of adsorbents that perform well in terms of CO/N_2_ selectivity and CO adsorption capacity, very little attention has been paid to their stability in humid and oxygen containing gas streams. In particular, no attention has been paid to the impact of competitive adsorption of water to the adsorption of CO. Regarding the performance test techniques, most research works use equilibrium adsorption setups that reports single-component adsorption isotherms for testing adsorption performance of the adsorbents. Sometimes, a single column dynamic adsorption setup is employed as a confirmatory technique to equilibrium adsorption. However, the transport and detailed kinetics of adsorption along with the adsorbate–adsorbent interaction at the molecular level are, thus, not tackled. The energy transport, that is to overcome the heat of adsorption, should be mentioned, which is certainly very impactful if one wants to employ chemisorption at scale. Though many adsorbents mentioned in literature show good adsorption capacity and selectivity towards CO with an IAST calculation, it is of importance to use realistic process conditions (for example, in a mixture of gases) for fair comparisons between different materials. It is obvious that an adsorbent will have a higher adsorption capacity at cryogenic temperatures than at room temperature and a similar argument could be made for the adsorption and desorption pressure. Furthermore, the material should be tested for stability and reproducibility over multiple adsorption–desorption cycles to quantify the dynamic performance, while now mostly only equilibrium adsorption isotherms and isosteric heats of adsorption are reported. Materials with a high equilibrium CO capacity do not necessarily possess a high working capacity as part of the CO is bound irreversibly under desorption process conditions. An example of this behavior is Ni-MOF-74, showing a significant difference in its equilibrium and dynamic CO capacity, as summarised in Table 3. A high binding affinity towards CO is beneficial for the selectivity as well, but this comes at the cost of adsorption reversibility with the conventional temperature or pressure swing processes. Attaining a significant working capacity of a material with a high affinity for CO thus requires more extreme process conditions, which increases the energy usage of the process and could affect the performance over time. Conventional swing processes expend energy in pressurising or heating the entire adsorption system, in order to change the chemical potential of the entire system. An improvement could potentially be made *via* adsorbents placed on electrically heated SiC supports. Adsorption processes would also become more efficient if they could selectively target and switch the adsorbate–adsorbent bond. Overall, other than developing novel adsorbents which could be used in realistic conditions, there is also a need to choose the right technique which can help to fully understand and quantify the actual value of adsorbents for CO purification at the industrial scale. Moreover, new process variables other than temperature or pressure, that can trigger the adsorption/desorption processes in a more energy-efficient way are also worth exploring.

### 3.3 Progress in absorption and membranes for CO-separation

Recent efforts in search for new absorbents are focused on the development of ionic liquids (IL) as a solvent for metal-complexes that selectively coordinate with CO absorbent. Using these ILs the solubility, stability, or environmental issues of COSORB could be resolved.^43,141–144^ Other than ILs being used solely as a solvent for complexes, ILs that intrinsically, selectively absorb CO are also being developed.^145,146^

The cuprate-based ionic liquid of David *et al.*^141^ reached a CO purity of 95% starting from a 1 : 1 mixture of CO and N_2_. Although the first results were promising, the sluggish mass transport rate typically shown by viscous ionic liquids and still relatively low CO capacity (2 mmol g^−1^) are hurdles that should still be overcome to develop a competitive process.^142^

Tao *et al.*^146^ developed an IL system based on carbanions which showed an exceptionally high CO solubility (0.11 mmol g^−1^) for a metal-complex free IL. However, this also shows that without the use of a transition metal complex the CO capacity of ILs is too low.

Viable absorption processes based on ILs require more work, but the plethora of possibilities in the IL toolbox offer perspective. Cui *et al.*^147^ sought to alleviate the mass transfer limitations by focusing on low viscosity deep eutectic solvents (DES). A DES consisting of 2-diethylaminoethanol chloride plus cuprous chloride (CuCl) plus ethylene glycol in a molar ratio of 1 : 1 : 4 showed the highest CO absorption capacity. However, this capacity is still only a modest 0.405 mmol CO/g at 1 bar and 293.3 K.

A novel absorption method was reported by Terry *et al.*^148^ in which they used electrochemistry to modulate the complexation of CO with CuCl/CuCl_2_. They note that Cu(ii) shows low affinity towards CO and Cu(i) shows high affinity and that by changing the Cu-ion oxidation state the electrolyte's ability to absorb CO can be altered. They pass the electrolyte solution containing the Cu(ii) through two porous carbon flow electrodes to produce Cu(i). This Cu(i) is then used to extract the CO from the feed gas stream in a hollow fiber membrane. The solution is then flowed through the two porous carbon flow electrodes oxidising the Cu(i) to Cu(ii). The CO is then recovered by once again passing the stream through a hollow fiber membrane. The electrolyte solution can then be reused by reducing the Cu(ii) again. By utilising this system they were able to change the CO and N_2_ pressure in the feed cylinder from 0.61 and 0.44 atm to 0.07 and 0.40 atm, respectively, while the CO and N_2_ pressure in the receiving flask changed from, respectively, 0.81 and 0.03 atm to 1.24 and 0.04 atm, at which point equilibrium was reached. The changes in pressure indicate that the electrochemically modulated absorption process selectively separated CO from N_2_. They note, however, that there are limitations to this technique. Firstly, low current densities are observed, resulting in a need for high surface area electrodes. Secondly, the utilisation efficiency of the Cu(i) is highly limited, with only 1 out of every 5 complexes being used in CO absorption at the pressures reported, while all Cu-ions undergo reduction and oxidation through the electrodes. Lastly, while the technique looks promising for further research, no further follow-up papers were published on electrochemical separation of CO.

To avoid the energy-intensive nature of the absorption process, supported liquid membranes that act as facilitated transport membranes, have also been explored for CO/N_2_ separation. Here the membrane liquid contains mobile carriers and is immobilized within the pores of a microporous support membrane that serves merely as a support layer.^149,150^ First Zarca *et al.*^151^ studied ionic liquid supported membranes, namely 1-hexyl-3-methylimidazolium chloride with CuCl as CO-carrier. Depending on the conditions CO/N_2_ selectivities of 2–4 were achieved, which is rather low. Moreover, upon addition of CuCl the CO permeability (at 303 K, 150 kPa pressure difference) increased only from 11.8 Barrer to 16.4 to Barrer. The limited solid-state facilitated transport was attributed to the low equilibrium constant of the complex formation, as well as the lower diffusivity of the CO–Cu(i) complex in the room temperature ionic liquid medium.

Feng *et al.*^152^ studied supported ionic liquid membranes using AgBF_4_ as carrier. This is inspired by membranes for ethylene/ethane separation where AgBF_4_ acts as carrier for ethylene *via* π-complexation.^153^ Also complexation of CO with Ag^+^*via* π-complexation *via* the mechanism described in Section 2.2.2 is expected.^154^ Feng *et al.* dispersed AgBF_4_ in 1-ethyl-3-methylimidazolium tetrafluoroborate [emim][BF_4_] as ionic liquid on a porous polyethersulfone membrane. Here the CO/N_2_ selectivity increased from ≈1 to ≈9 going from no AgBF_4_ to 0.3 : 1 AgBF_4_:[emim][BF_4_] (measured at 293 K, 0.45 MPa). The permeability, however, decreased dramatically with increasing AgBF_4_ content, from ≈300 Barrer to ≈20 Barrer.

Later, Kim *et al.*^155^ achieved a slightly better result *via* combining AgBF_4_ with [bmim][BF_4_] (1-butyl-3-methylimidazolium tetrafluoroborate) and a comblike copolymer poly(2-hydroxypropyl-2-(methacryloyloxy) ethyl phthalate-*co*-acrylic acid), which were coated together on a polyethersulfone membrane. The highest achieved CO/N_2_ selectivity was 16.2 and the CO permeance was 2.1 GPU (gas permeance units). A membrane containing the ionic liquid but not AgBF_4_ for comparison was unfortunately not studied.

Solid state facilitated transport membranes have also been investigated. In 2019, Park *et al.*^156^ reported on AgBF_4_ and MgO nanosheets embedded in poly(ethylene glycol) behenyl ether methacrylate-poly(methacrylic acid). The latter polymer was chosen as the carboxylic acids groups would minimise CO_2_ permeance, and the MgO nanosheets should stabilize AgBF_4_. A CO permeance of 79 GPU and separation performance of CO/N_2_ of 14.7 and CO/CO_2_ of 12 were achieved.

**Fig. 12:**
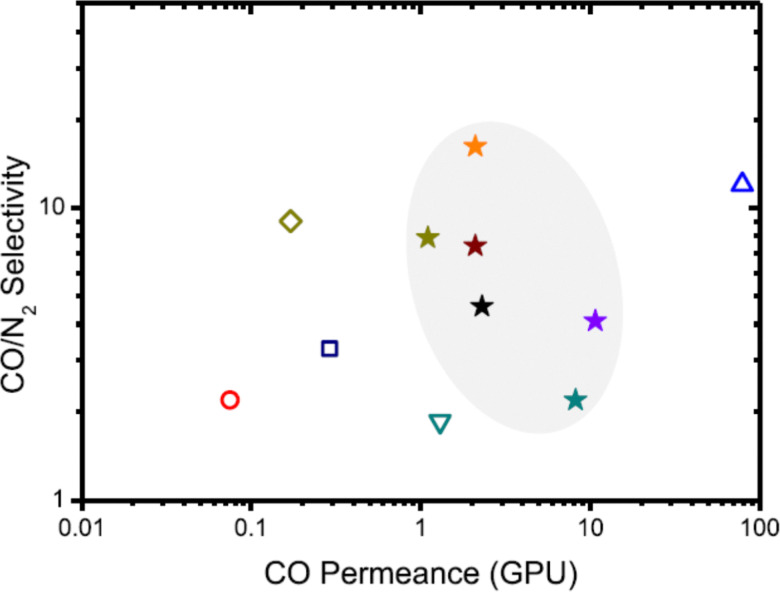
Plot of CO/N_2_ selectivity *versus* CO permeance for various membranes. Star symbols (*) represent ref. 155. The blue triangle represents ref. 156, the other symbols represent ref. 151, 152, 158 and 159. Reprinted from ref. 155, Copyright (2021) with permission from Elsevier.

The most recent example of a solid state facilitated transport membrane is a system were AgBF_4_ and Ag nanoparticles in metal–organic frameworks MIL-101 are proposed to work as dual carriers.^157^ The materials are dispersed in a comb copolymer poly(glycidyl methacrylate)-*co*-poly(oxyethylene methacrylate) deposited onto a porous polysulfone support. Upon addition of 10 weight percent of Ag@MIL-101 (compared to the copolymer) the CO/N_2_ selectivity increased from 3.3 of 11.8, and the CO permeance from 24.8 GPU to 30.7 GPU. Fig. 12 provides an overview of the different reported CO/N_2_ selectivities *versus* CO permeances for several membranes.

In conclusion, the intrinsic energy intensive nature of liquid phase absorption processes and complex stability issues render them incompatible with recent sustainability ambitions. The use of ionic liquids and deep eutectic solvents makes them less harmful for the environment, but due to the relatively low CO absorption capacity and the high viscosity of the ILs, the high energy requirement for regeneration remains. Promising results have been achieved *via* membranes, an intrinsically less energy-intensive separation technology, based on facilitated transport *via* a carrier that selectively binds CO. However, these are based on Cu(i) and Ag(i) complexes and are studied in a water and O_2_ free environment, probably because these complexes are likely unstable in presence of these molecules. It would be interesting to see future work on facilitated transport membranes particularly for wet flue gasses.

## 4 Traditional *versus* emerging CO sources

### 4.1 Traditional CO sources from industrial processes

Industrially, carbon monoxide can be produced by partial oxidation and steam reforming of carbonaceous feedstocks (*e.g.*, natural gas, steam reforming: CO: 15.5%; N_2_: 0.2%; CO_2_: 8.2%; H_2_: 75.5%; CH_4_: 0.5%; dry basis), as well as *via* coal gasification (coal gasification: CO: 59.4%; N_2_: 0.6%; CO_2_: 10.0%; H_2_: 29.4%; other species: 0.6%; dry basis) (Table 4^160^). As a co-product mainly along with hydrogen, carbon monoxide gases are then separated and purified by pressure swing adsorption and/or cryogenic distillation.^161^ CO separated from these product streams can further be used as chemical building block in various chemical processes.

**Table 4:** Typical excess gas compositions from industrial processes^1,6,160^

	Blast furnace gas	Coke oven gas	Basic oxygen furnace gas	COREX® export gas	FINEX® export gas
Gas production	∼900 m^3^ per ton steel	∼50 m^3^ per ton steel	∼50 m^3^ per ton steel	—	—
*P*_abs_ [MPa]	Up to 0.3	—		0.1	0.1
*T* [°C]	100–200	800		40	40
Gas component	Vol (%)				
CO	20–28	4–7	55–80	40–50	30–50
N_2_ + Ar	50–55	0–10	8–26	2	10
CO_2_	17–25	1–3	10–18	25–35	25–45
H_2_	1–5	39–65	2–10	15–25	12–25
CH_4_	—	20–42	—	2	1.5
C_*x*_H_*y*_	—	2–8.5	—	—	—
Other species	Water vapour, H_2_S, sulfur, cyanide compounds	Water vapour, H_2_S, benzene, toluene, xylene, ammonia, other hydrocarbons, sulfur compounds	Water vapour, H_2_S, sulfur compounds	Water, H_2_S	Water H_2_S

Meanwhile, large amounts of CO can also be found in the off gases of metallurgic processes, especially in the blast furnace gas (BFG: 20–28% of CO), basic oxygen furnace gas (BOFG: 55–80% of CO), and to a smaller extent in the coke oven gas (4–7%) of integrated steel mills (Table 4^6^). However, most attention is paid to the separation of CO_2_ from these off-gases and very little information is available in open literature regarding the industrial separation of CO from BFG or BOFG. Only the PSA process named “COPISA” is claimed to be used for the separation of CO from the BFG process by Kawasaki Steel Corporation and Osaka Oxygen Industries LTD (Section 2.4.4.1). In most cases, CO is currently not separated from these off-gases, the main bottleneck being the difficult separation of CO and N_2_. Instead, it is being oxidised to CO_2_ and emitted. Direct CO_2_ emission from steel and iron manufacturing accounts for 5–7% of all man-made CO_2_ emissions in the world.^162^ Roughly one third of this CO_2_ is created *via* oxidation of CO. Preventing avoidable CO_2_ by capture of CO, hence, will have a significant impact in achieving carbon-neutrality.

Coal gasification and iron making off-gases are typically rich in carbon monoxide and show a low syngas ratio. Smelting reduction processes, such as COREX and FINEX, are a newer iron making technology.^160^ With less impurity and lower nitrogen content (<10%) than blast furnace gas, COREX (with 40–50% of CO) and FINEX (with 30–50% of CO) export gases seem to be attractive as alternative feedstock for the generation of CO. (Table 4^1^) However, there are no reports concerning plants in operation for the utilisation of these two export gases as syngas in chemical industry nor are there reports regarding separating CO from these gases.

### 4.2 Emerging non-traditional CO sources

**Fig. 13:**
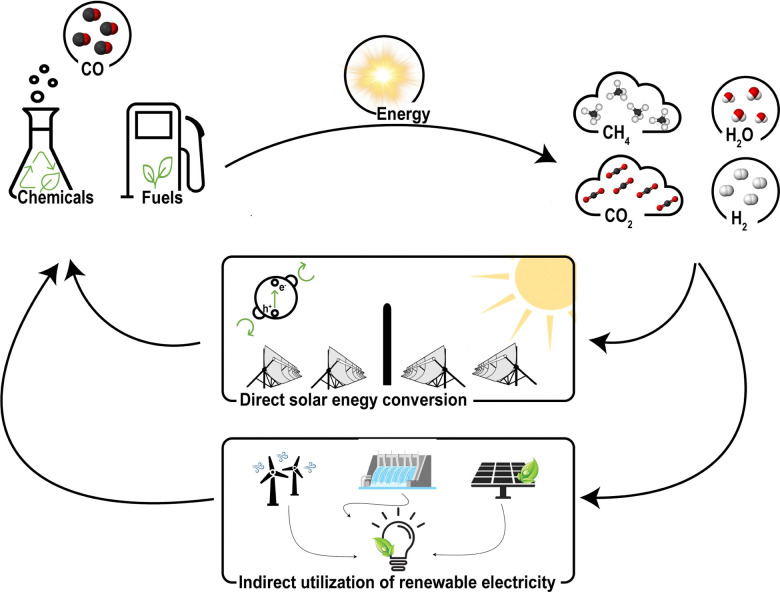
Schematic of emerging processes utilising renewable energy for the conversion of carbon dioxide enabling the development of carbon-neutral cycles.

With the transition towards a more sustainable chemical industry and the need for utilisation of carbon dioxide as an alternative carbon source, a variety of new carbon-chemical processes will emerge in the near future (Fig. 13). Renewable electricity or direct solar light capture will be used to power the envisioned processes, ultimately leading to a carbon neutral cycle.^163^ Similar to the addressed traditional fossil-based sources, CO will likely be present in a complex product stream and purification or recovery will be essential to enable carbon monoxide utilisation. Here we outline emerging processes that are foreseen as possible CO sources and discuss their status, *i.e.*, technology readiness level (TRL). Moreover, the required energy input, the expected product compositions, the associated challenges for CO separation, and general limitations of the technologies will be briefly addressed.

Among the explored strategies, direct solar energy utilisation is of great interest. Here, additional energy input is not required and with an average solar flux of 175 W m^−2^ sufficient energy is made available at the earth's surface within 2–3 hours covering the annual worldwide energy consumption.^163,164^ Direct solar utilisation is classified into solar thermochemical and photon-driven processes. The latter direct photo-driven CO_2_ reduction processes leverage the photon energy directly to drive a redox reaction, for example, using a semiconductor with appropriate valence and conduction band positions. Direct photo-driven CO_2_ electrolysis eliminates the intermediate step of transferring electricity or converting light into heat, offering a thermodynamic advantage. Still, the kinetic overpotentials of the CO_2_ reduction reaction and concomitant oxygen evolution imply the use of semiconductor materials with band gaps larger than 2.5 eV and/or usage of multifunction photovoltaic cells.^165,166^ Photocatalytic processes are additionally hampered by internal and surface recombination as well as limited stability of photoelectrodes. Hence, a trend towards protecting or even separating photovoltaic layers from electrocatalytic layers has been ongoing, which has blurred the interface between direct photo-driven CO_2_ electrolysis and CO_2_ electrolyzers indirectly driven by solar energy *via* the power grid.^167^ While for these indirect solar-to-CO conversion processes efficiencies >5% have been reported,^168^ the direct photocatalytic or photoelectrochemical CO_2_-to-CO conversion has arguably the lowest TRL level (TRL 3) among the technologies.^166^

Solar thermal approaches are more advanced in their development. In fact, power generation using solar towers or dishes is currently applied on larger-scale and will potentially allow for a straightforward implementation of solar reactors.^169,170^ Generally solar thermochemical processes make use of concentrated solar heat to drive a thermochemical cycle. In a first step, generated solar heat enables reduction of metal oxides. Subsequent oxidation of reduced metal oxides in a nonsolar exothermic reaction by CO_2_ and/or water facilitates formation of CO and/or H_2_. The theoretical oxide-dependent (*e.g.*, CeO_2_) solar-to-fuel efficiency of solar thermal processes is typically 20% therefore being on par with the break-even point for cost competitive production of direct solar-driven CO_2_ utilisation. Small prototypes for water splitting and/or CO_2_ utilisation are already in operation (TRL 4–5).^171^ Thus, the final product stream of solar-thermal CO_2_ conversion processes will at least be a mixture of CO and CO_2_, or CO/CO_2_/H_2_ and water in the case of concomitant water splitting. Hence, selective product separation technologies for practical CO_2_ to CO conversion is needed.^170^ Still, implementation of solar thermal processes is hampered by the insufficient durability of available metal oxides and new materials with fast oxygen exchange kinetics and high stability are required for commercial success.^170^

In contrast to above mentioned technologies, electrochemical and plasma conversion rely on electricity. Their reliance on electricity might contradict the purpose of greenhouse gas mitigation. However, the global transition to solar and wind energy implies that both electrochemical and plasma conversion technologies can be considered carbon neutral.^172–175^ Amongst other processes, plasma approaches allow for pure splitting of CO_2_ to carbon monoxide and oxygen. Various versions of the specific implementation of the plasma technology exist (for a detailed understanding of the available plasma technologies the reader is referred to Snoeckx *et al.*^171^), however, all of them have in common that the CO_2_ conversion efficiency is sacrificed by energy efficiency and *vice versa*. So far only gliding arc and microwave plasma are capable of reaching energy efficiencies of >60% with CO_2_ conversions of up to 40%, whereas carbon dioxide conversions in the range of 40–90% are only achievable at energy efficiencies below 40%. The TRL level of plasma technologies for CO_2_ splitting is considered to be TRL 3–4. Plasma technologies also allow for CO_2_ conversion with hydrogen (or water) and dry-reforming of methane. For both processes the product mixture is complex compared to pure CO_2_ splitting and feed gas ratios largely influence conversion rates and energy cost/energy efficiency.^174^ Stil, conversion efficiencies for dry-reforming of methane in plasma reactor are often reported to exceed 70% at energy efficiencies of >50%.^171^

At present, most scientific studies for CO_2_ conversion are electrochemical, either at high temperature or low temperature operation, both at a slightly higher TRL compared to plasma technologies or solar driven conversion. These technologies have been demonstrated at tens of cm^2^ scale^175–177^ and are being developed by several companies (Siemens, Sunfire, Opus12, Shell, *etc.*).^178^ The designs for the electrochemical routes tap from the more developed electrochemical processes of, *e.g.*, water electrolyzers, fuel cells and chlorine production. Hence, based on the available industrial experience it is expected that direct or indirect electrochemical CO generation is closest to industrial application and will be addressed in more detail in Section 4.3.

### 4.3 CO capture after electrochemical reduction of CO_2_

**Fig. 14:**
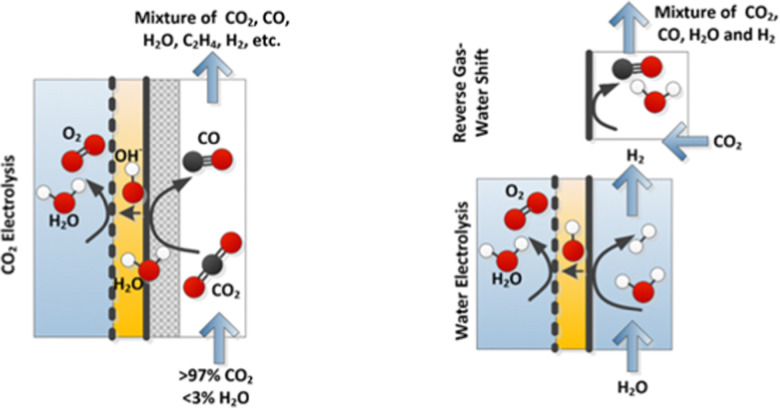
Direct CO_2_ to CO conversion (left), in this case in a reactor with a liquid anode (zero-gap) and CO_2_ reduction at the vapour-fed cathode, and indirect CO_2_ conversion (right), *via* water electrolysis and Reverse Gas–Water Shift Reaction (RGWSR). For simplicity, reaction stoichiometry is ignored.

As an alternative to obtaining syngas from steam reforming, syngas can be produced from renewable resources using electrochemistry. Two electrochemical routes for obtaining CO are available (Fig. 14): (1) indirect CO_2_ conversion *via* electrochemically produced hydrogen coupled with reverse water–gas shift reaction or (2) direct electroreduction of CO_2_. Both routes provide a pathway to utilise CO_2_ for hydrocarbon fuels and carbon-based chemicals thereby closing the carbon cycle. Regarding fuels, producing synthetic hydrocarbon fuel is typically less energy efficient than producing green hydrogen as an energy carrier, but hydrocarbon products are liquid or can be liquefied.^179^ The EU predicts that up to 4% of their consumed energy is transported *via* e-liquids (and roughly 5% *via* e-gas) by 2050.^180^ In addition, currently 14% of our fossil oil is converted into petrochemicals^181^ and needs to be replaced with a carbon-neutral alternative when completing the energy transition towards renewables. Because carbon monoxide is a versatile precursor for many hydrocarbon-based fuels and chemicals, electrochemical routes for synthetically produced CO have gained attention.

Synthetic carbon monoxide can be obtained using mature development technology *via* water electrolysis driven by renewable energy (producing green hydrogen) and applying the reverse water–gas shift reaction (RWGSR) to a mixture of hydrogen and CO_2_ gas (Fig. 14 right). This endothermic RWGSR, for example using Fe-, Cu- or Co-based catalysts, is favored at elevated temperatures to achieve high conversion to CO with fast kinetics.^182,183^ Typically, 40–70% of the CO_2_ can be converted. Side products are possible, such as methane, but the selectivity towards carbon monoxide is high (>90%) at elevated temperatures.^184^ Hence, carbon monoxide separation technologies for this route should deal with a relatively high CO concentration in a matrix of H_2_, CH_4_ and CO_2_.

A more challenging separation may be required for the carbon monoxide produced from direct CO_2_ electroreduction (Fig. 14 left). A multitude of routes has been studied for direct electrochemical CO_2_ conversion, including solid oxide electrolysis, molten carbonate electrolysis, low-temperature aqueous-dissolved CO_2_ reduction and low temperature vapour-phase CO_2_ reduction. From these technologies for direct CO_2_ electroconversion, the latter has been developed in medium scaled cells by startup companies and industry. Scientific literature also shows extensive studies on the low temperature vapour-phase CO_2_ reduction, which has achieved higher current densities (>1 A cm^−2^) owing to the high diffusivity of CO_2_ in gas phase.

**Fig. 15:**
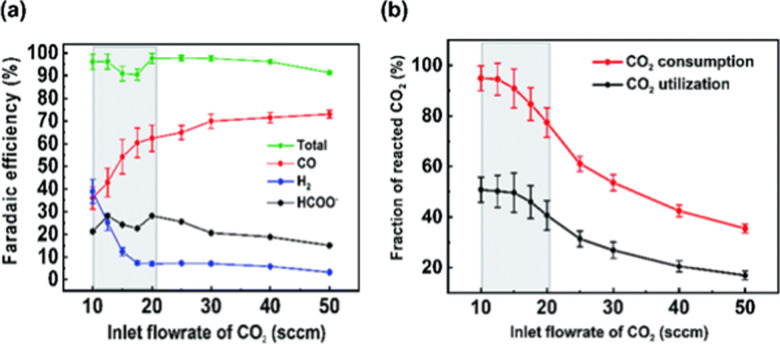
Faradaic efficiency of products for various inlet flow rates performed at a current density of 200 mA cm^−2^. (b) CO_2_ utilisation and CO_2_ consumption for different inlet flow rates at 200 mA cm^−2^. Greyed regions represent trade-offs between utilisation and selectivity. CO_2_ consumption is always higher than CO_2_ utilisation because CO_2_ is crossing over to the analyte. Reproduced from ref. 186 with permission from the Royal Society of Chemistry, copyright 2021.

In the field of low-temperature (vapour-fed) electrochemical CO_2_ reduction, most efforts have ignored the single pass conversion fraction of CO_2_ to CO, and have fed excess of CO_2_ to allow focusing on material development, high current density, energy efficiency and faradaic efficiency. Therefore, the concentration of CO in the product stream is typically very low (<10%) in scientific studies, implying large efforts for the separation process. Some research has studied direct CO_2_ electroreduction with a high fraction of CO_2_ to CO conversion. However, still 40–80% of the CO_2_ remains unreacted in a single pass conversion step, even when the CO_2_ feed rate is optimised.^185–187^ Moreover, a wide variety of products can be produced by CO_2_ electroreduction (carbon monoxide, ethylene, hydrogen, ethanol, formic acid). In particular, to minimise the CO_2_ concentration in the outlet gas, the CO_2_ supply must be limited, which increases the relative hydrogen production and the local pH at the electrode, favoring CO_2_ conversion to formic acid (Fig. 15). Two strategies can be pursued to increase CO concentrations. First, the CO_2_ to CO single pass conversion fraction can be optimised, compromising the faradaic efficiency and energy efficiency, but creating a product stream with concentrated (>50%) CO. Alternatively, a low CO concentration can be accepted if a highly selective separation technology is implemented to allow recycling of unreacted CO_2_. Hence, advancing separation technology of CO will largely impact the CO_2_ electroreduction possibilities.

## 5 Conclusions and outlook

Despite CO being a key chemical building block in the chemical industry (*e.g.*, in Fischer Tropsch synthesis), advancements in its separation are still very much needed. On one hand, there are traditional technologies that produce process streams high in CO content, like metallurgic flue gasses, where CO is barely recovered, but solely used for its caloric value *via* oxidising it to CO_2_. This constitutes 2.7% of manmade CO_2_ emissions.^188^ On the other hand emerging processes produce complex product mixtures with variable CO concentrations. Even for the emerging process that are optimized for CO production, *i.e.*, electrochemical carbon dioxide reduction to CO, product streams will likely contain impurities such as water, carbon dioxide, and (traces) of hydrogen, exemplifying the urgency of further research and industrial implementation of energy efficient CO separation processes. In fact, carbon neutrality and the electrification of the chemical industry poses a need for research and development of efficient CO separation to be directly integrated into emerging and existing processes.

With regard to metallurgic off-gases, the main focus is on the separation of CO and N_2_, which are hard to separate due to their very similar physical properties. Most commercial processes are thus based on chemisorption of CO *via* CuCl complexes. Among these, the absorption process (COSORB) shows the best performance in terms of CO purity and recovery, but is also very energy consuming due to its heating and cooling cycles. In contrast, adsorption processes are more energy-efficient, but high CO purity can only be achieved by compromising recovery. Particularly, for the separation process relying on strong physisorption (based on Na^+^ Mordenite), instead of chemisorption, the loss in recovery is particularly large (98% purity with 45% recovery). Moreover, all these processes require pretreatment to remove water to avoid competitive adsorption and/or decomposition of the CuCl complexes, which also comes at an energetic cost. The limited degree to which these processes are actually implemented to separate CO from metallurgic off-gases, indicates that a sufficiently satisfactory technology has not been developed so far.

The last decades has thus seen significant research effort in developing new CO separation processes. In the field of absorption, to mitigate the environmental risks associated with a solvent based absorption process, current research is focused on solvents with low volatility, like ionic liquids and deep eutectic solvents. However, the limited CO absorption capacities reached, combined with the ever-present high energy needs for the heating and cooling cycles, and mass transfer limitations of ionic liquids, poses the question of the expected impact of this direction of research. As such the research effort in finding new adsorbents was much larger. In the last decades, many different types of porous solids have been investigated, from activated carbon, zeolites, mesoporous silica and alumina to metal–organic frameworks. Most of these studies are based on the doping of these materials with transition metal complexes capable of π-complexation with carbon monoxide to achieve chemisorption. Most typically, like in the commercial ad/bsorption processes, CuCl is used, leading to CO/N_2_ selectivities ranging from 35 to 66. Metal–organic frameworks themselves can also show high CO/N_2_ selectivity if they contain coordinatively unsaturated transition metal sites capable of π-complexation with CO, especially for Cu(i), Co(ii), Ni(ii) and Fe(ii) high CO/N_2_ selectivities, ranging from 46 to 216, have been achieved. Particularly intriguing adsorbents (*e.g.*, Fe_2_Cl_2_(bbta)^130^) based on a cooperative spin crossover mechanism accompanying CO adsorption have been found, which show a step CO adsorption isotherm, meaning that only a very small change in CO partial pressure would be needed to swing between adsorption and desorption. Such a mechanism has large potential to be energy-efficient.

The available studies generally provide an ideal CO/N_2_ selectivity, the CO-adsorption capacity, as well as the adsorption enthalpy. Regularly adsorption capacities as high or higher than those of the commercial CO-adsorbents (>3 mmol g^−1^) are measured, and in some cases also the dynamic capacity (defined as change in equilibrium adsorption between 0.1 and 1 bar at measured temperature) is above this value. Competitive adsorption of CO and N_2_, however, is scarcely measured, but is needed to make a realistic assessment of the selectivity. Breakthrough profile measurements would, moreover, allow for assessment of whether significant changes in the amount of adsorbed CO can be obtained within a reasonable temperature or pressure range, thus improving the regeneration potential, as well as the stability of performance over many adsorption/desorption cycles. Performing this work in humid streams would additionally allow to assess both hydrolytic stability as well as competitive CO, H_2_O adsorption. Currently, breakthrough studies and studies on wet gasses have rarely been conducted. It is very likely that the adsorbents based on doped CuCl have limited stability in wet gasses. The metal–organic frameworks containing coordinatively unsaturated transition metal sites may be hydrolytically stable, depending on the framework.^189^ Whether CO can be competitively absorbed in the presence of water would need to be investigated. In general, with regard to industrial applicability, it is also important that the adsorbents are tested with respect to (1) resistance to attrition (especially where circulating and moving bed process implementations are considered), (2) heat transport, as adsorption is an exothermic process, and (3) corrosion sensitivity for the feed stream by including impurities such as SO_x_, NO_x_ and HCl.

Considering the importance of CO/CO_2_ separation among emerging technologies perceived as being important to achieve defossilisation, *e.g.*, electrocatalytic reduction of CO_2_ to CO, it is vitally important to include CO capture of CO from streams rich in CO_2_ and, again, ideally wet streams. Nevertheless, only a subset of the available studies include determination of the CO/CO_2_ ideal selectivity revealing the need to steer research efforts in this direction. Due to the stronger physisorption of CO_2_ compared to N_2_, typically the CO/CO_2_ is an order of magnitude lower than the CO/N_2_ selectivity and is especially poor in materials with small micropores where the physisorption of CO_2_ is strong. Considering the emerging importance of CO/CO_2_ separation within the frame of defossilisation, this would deserve more attention in future research, ideally through breakthrough profile measurements of wet gas mixtures.

A limited amount of work has been performed regarding CO/N_2_ separation *via* membrane separations. Regarding CO/N_2_ membrane separation, some promising results based on facilitated transport membranes containing transition metal complexes (most notably AgBF_4_) have been achieved. Considering that the scope of work is very limited and that membrane separations operate generally in an energy efficient manner, this might be an area where significant progress could be made. Again, ideally these membranes are also assessed for wet streams, and include CO/CO_2_ separation. At the present, as they are based on Ag(i) and Cu(i) carriers they are unlikely to be stable in wet streams.

The emerging separation problem of separating CO from CO_2_/H_2_O/H_2_ in the frame of electrochemically produced CO, might alternatively be tackled *via* removing CO_2_ from the mixture for feedback to the reaction mixture *via* existing adsorbents and membranes for CO_2_ removal. Thus in absence of transition metal cations, materials with a high CO_2_/CO selectivity based on the often stronger physisorption and solubility of CO_2_ compared to CO are useful. Still, any adsorbent or membrane for the removal of CO should be compared techno-economically against processes based on CO_2_ selective materials.

These identified challenges provide a guidance for future research. For environmental concerns, meaning the need for energy-efficient separation processes, the likely focus will be on adsorption and membrane separations. With regard to adsorbents, strong CO_2_ adsorption should be avoided. Through a large research effort in the last decades, an understanding of what structural features lead to strong CO_2_ adsorption has emerged.^117,190^ This means that amine functional groups and ultramicropores should in general be avoided to achieve high CO/CO_2_ selectivity. All well-performing adsorbents capable of CO/N_2_ separation seem to be based on chemisorption *via* π-back bonding with transition metals. The stability of the redox state of the transition metal ion is the first concern for compatibility in the presence of H_2_O and O_2_. Cu(i) and Fe(ii) have been the most studied, but they are unstable in these conditions. Potentially, *via* applying a sufficiently negative electrical bias to an adsorbent in an electrochemical cell, the lower oxidation state of Cu(i) and Fe(ii) could be stabilised. Alternatively, the few adsorbents based on Co(ii) and Ni(ii) have an equally adequate adsorption enthalpy and CO/N_2_ selectivity, while these elements are notably more stable in this oxidation state. Studying these materials for their water and oxygen stability is necessary, as well as developing new adsorbents based on these ions.

Many streams from which we want to separate CO are also humid streams. Ideally, the CO adsorbent can selectively adsorb CO, also at a relative humidity (RH) of relevance from a process technology point-of-view, *e.g.* 40% RH. Competitive adsorption of CO over water *via* π-back bonding with transition metals is possible. For example', CO binds preferentially over water to the Fe(ii) of haemoglobin. Although, that is if we consider a single adsorbed H_2_O molecule *versus* a CO molecule. For haemoglobin this is a realistic situation, due to the hydrophobic pocket in which the Fe(ii) site is embedded. However, when the Fe(ii) porphyrin motif is surrounded by water, the extent to which hydrogen bonds need to be broken for the adsorption of a molecule of CO, probably makes competitive CO adsorption energetically unlikely. Hence, a search for microporous materials with motifs for CO chemisorption in an otherwise hydrophobic structure in which pore filling by water cluster formation does not take place at low to modest relative humidities (*e.g.*, 40%RH) is needed. Some structure-property relationships which lead to such water sorption behaviour are discussed in the literature for activated carbons^191^ and metal–organic frameworks,^192,193^ and can be applied in the search for new CO-adsorbents. Similar material design considerations as outlined here for adsorbents, can also be used for the development of new and more stable facilitated transport membranes. The scant research on membranes for separation of CO *via* facilitated transport means that there is a large potential for progress in this field.

In short, while significant progress has been achieved in research literature regarding the separation of CO, we highlight the importance and the lack of investigating especially the separation of CO/N_2_ and CO/CO_2_ from humid streams in realistic dynamic conditions.

## Author contributions

LS: literature search, Wr: writing, Ed: editing. XM: LS & Wr: 1, 2.3, 2.4, 3.2, 4.1; Ed: 2.1; DG: LS & Wr: 2.4; BM: LS & Wr: 4.2; Ed.: 4.3 & 5; MV: LS & Wr: abstract, 2.4.5, 3.2.2, 3.2.5, 3.3, 5; Ed: whole manuscript; FK: Ed: 1, 2.1–2.4, 3.1; SB: LS: 1, 2.1–2.3, 3.2; Wr: 1; Ed; 1, 2.1–2.3, 3.1–3.2, 5; RH: LS: 2.1, 3.2 Wr: 3.2; DV: LS & Wr: 4.3, Ed: 4.2; JA: LS & Wr: 2.2, 2.3, 3.2, 3.3; Ed: whole manuscript; SP: LS & Wr: 3.1; CS: LS & Wr: 2.1, 2.4.1; BE: LS & Wr & Ed: 2.1, 2.4.1, 3.1.

## Conflicts of interest

There are no conflicts to declare.

## Supplementary Material
